# Genomic sequencing and functional analyses identify MAP4K3/GLK germline and somatic variants associated with systemic lupus erythematosus

**DOI:** 10.1136/annrheumdis-2021-221010

**Published:** 2021-10-05

**Authors:** Huai-Chia Chuang, Wei-Ting Hung, Yi-Ming Chen, Pu-Ming Hsu, Jeng-Hsien Yen, Joung-Liang Lan, Tse-Hua Tan

**Affiliations:** 1 Immunology Research Center, National Health Research Institutes, Zhunan, Taiwan; 2 Division of Allergy, Immunology and Rheumatology, Taichung Veterans General Hospital, Taichung, Taiwan; 3 Department of Internal Medicine, Kaohsiung Medical University Hospital, Kaohsiung Medical University, Kaohsiung City, Taiwan; 4 Rheumatology and Immunology Center, China Medical University Hospital, Taichung, Taiwan; 5 College of Medicine, China Medical University, Taichung, Taiwan; 6 Department of Pathology and Immunology, Baylor College of Medicine, Houston, Texas, USA

**Keywords:** lupus erythematosus, systemic, autoimmune diseases, inflammation

## Abstract

**Objectives:**

MAP4K3 (GLK) overexpression in T cells induces interleukin (IL)-17A production and autoimmune responses. GLK overexpressing T-cell population is correlated with severity of human systemic lupus erythematosus (SLE); however, it is unclear how GLK is upregulated in patients with SLE.

**Methods:**

We enrolled 181 patients with SLE and 250 individuals without SLE (93 healthy controls and 157 family members of patients with SLE) in two independent cohorts from different hospitals/cities. Genomic DNAs of peripheral blood mononuclear cells were subjected to next-generation sequencing to identify GLK gene variants. The functional consequences of the identified GLK germline or somatic variants were investigated using site-directed mutagenesis and cell transfection, followed by reporter assays, mass spectrometry, immunoblotting, coimmunoprecipitation, and in situ proximity ligation assays.

**Results:**

We identified 58 patients with SLE from Cohort #1 and #2 with higher frequencies of a somatic variant (chr2:39 477 124 A>G) in GLK 3′-untranslated region (UTR); these patients with SLE showed increased serum anti-double-stranded DNA levels and decreased serum C3/C4 levels. This somatic variant in 3′-UTR enhanced GLK mRNA levels in T cells. In addition, we identified five patients with SLE with GLK (A410T) germline variant in Cohort #1 and #2, as well as two other patients with SLE with GLK (K650R) germline variant in Cohort #1. Another GLK germline variant, A579T, was also detected in one patient with SLE from Cohort #2. Both GLK (A410T) and GLK (K650R) mutants inhibited GLK ubiquitination induced by the novel E3 ligase makorin ring-finger protein 4 (MKRN4), leading to GLK protein stabilisation.

**Conclusions:**

Multiple GLK germline and somatic variants cause GLK induction by increasing mRNA or protein stability in patients with SLE.

**Key messages**

**What is already known about this subject?**

- Both heritable and environmental factors are linked to systemic lupus erythematosus (SLE) pathogenesis. MAP4K3 (GLK) overexpression in T cells induces interleukin (IL)-17A production and autoimmune responses. The frequency of GLK overexpressing T cells is correlated with severity of human SLE.

**What does this study add?**

- GLK 3′-untranslated region (UTR) (T635C), GLK 3′-UTR (A644C), GLK (A410T) or GLK (K650R) variant-induced GLK overexpression through the stabilisation of GLK mRNAs or proteins may be involved in SLE pathogenesis. To our knowledge, this is the first identification of the novel E3 ligase MKRN4 that induces GLK protein degradation. GLK (A410T) and GLK (K650R) variants block MKRN4-induced Lys48-linked ubiquitination of GLK.

**How might this impact on clinical practice or future development?**

- SLE is difficult to be diagnosed at the early stage. Our findings suggest that individuals harbouring GLK variants or MKRN4 dysregulation/mutation accompanied by other risk factors could be at high risk for SLE.

## Introduction

Systemic lupus erythematosus (SLE) is a chronic, complex and systemic autoimmune disease with multiorgan damages.^1^ Both heritable and environmental factors are linked to SLE pathogenesis.^2–4^ About 95% of patients with SLE display an induction of antinuclear autoantibodies.^5^ Increased serum anti-double stranded DNA (dsDNA) autoantibody levels are correlated with enhanced SLE disease activity, whereas serum complement C3/C4 levels are inversely correlated with SLE disease activity.^6^ Autoantibodies trigger complement responses and amplify inflammation, leading to multiorgan damages in patients with SLE.^7^ Moreover, Th17 (CD4^+^ IL-17A-producing T) cells contribute to autoimmune responses by recruiting macrophages and facilitating B-cell activation.^8^

Makorin ring-finger protein 4 (MKRN4, also named MKRN4P, RNF64, ZNF127L1) is a putative ubiquitin-protein E3 ligase, which was thought to be a pseudogene prior to 2010 due to the deficiency of MKRN motif, the lack of introns and the presence of a poly-A region.^9 10^ In 2011, the deposited full-length MKRN4 mRNA sequence was deposited (NCBI accession number: NG_004713.4).^11^ MKRN4 protein in fact, similar to other MKRN family members, contains four conserved C3H domains, one conserved MKRN Cys-His motif, and one conserved C3HC4 ring-finger domain. The MKRN family of ubiquitin E3 ligases includes MKRN1, MKRN2, MKRN3 and MKRN4.^9 12^ MKRN4 shares 81% amino acid identity with MKRN1, 46% amino acid identity with MKRN2 and 52% amino acid identity with MKRN3.^9 12^ To date, the functions and targets of MKRN4 remain completely unknown.

The serine/threonine kinase MAP4K3 (also named GLK) directly interacts with and phosphorylates PKCθ in T cells, resulting in T-cell activation.^13^ GLK overexpression in murine T cells induces IL-17A production and T-cell hyperactivation, leading to autoimmune inflammatory diseases.^14^ Moreover, GLK is overexpressed in T cells in patients with autoimmune diseases including SLE^13 15–18^; GLK-overexpressing T-cell population is correlated with the disease severity of patients with SLE.^13 17^ To date, the mechanism of GLK overexpression in patients with SLE remains unclear. Here we explored whether GLK genetic variants occur in patients with SLE by next-generation sequencing using two independent cohorts of patients with SLE from different cities.

## Results

### Both GLK somatic and germline variants occur in patients with SLE

**Figure 1:**
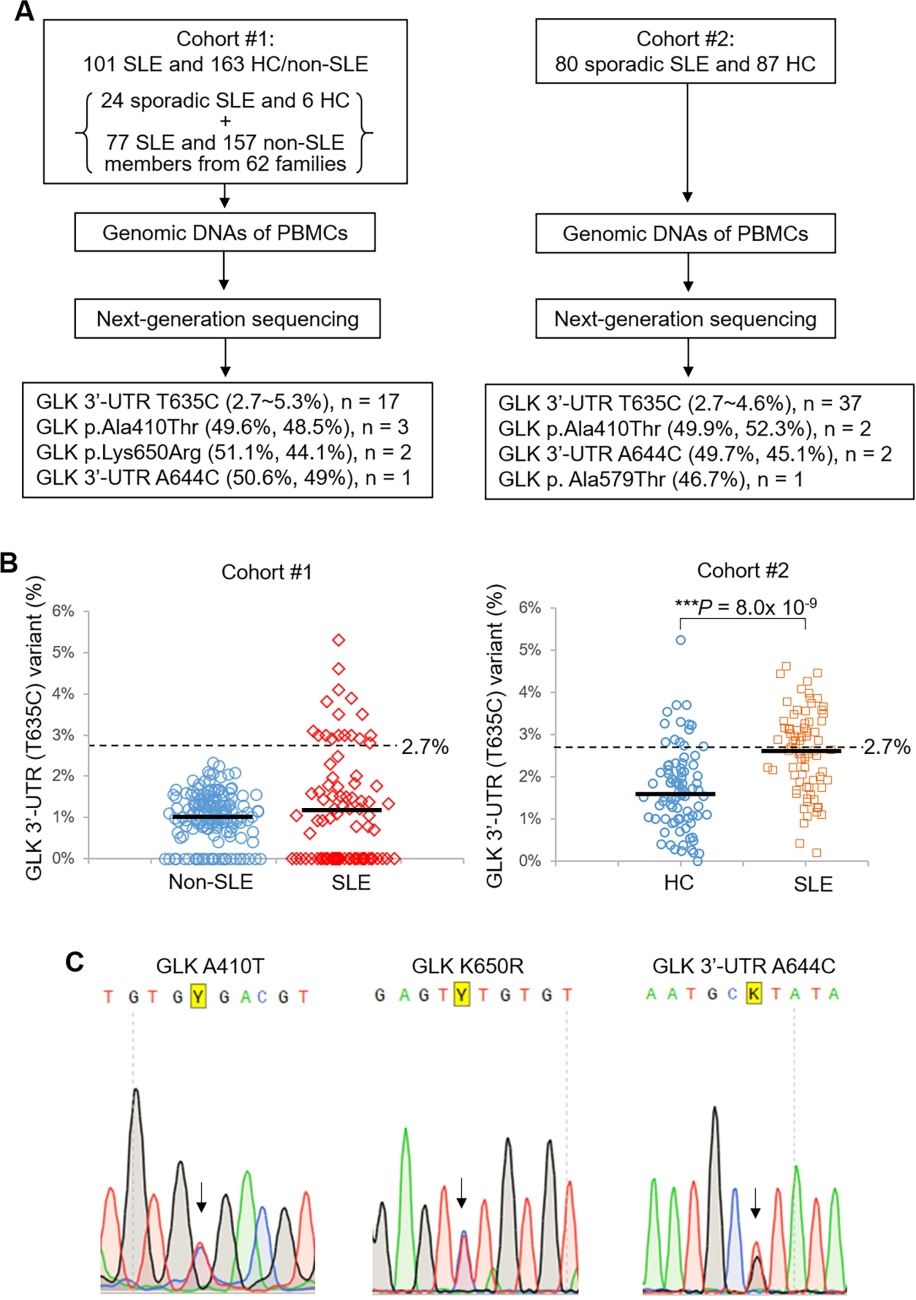
GLK somatic and germline variants occur in PBMCs of SLE patients. (A) Schematic diagram of the screening design to identify GLK gene variants in patients with SLE by next-generation sequencing. (B) The variant frequency of GLK 3′-UTR (T635C) variant in 163 individuals without SLE (6 HCs and 157 members without SLE from individual families) and 101 patients with SLE (sporadic and familial) from Cohort #1 (left panel). The value 2.7% was mean plus 3SD (1.0% + 3 × 0.58%=2.74%) of 3′-UTR (T635C) variant frequencies in the group without SLE. The frequency of GLK 3′-UTR (T635C) variant in 87 HCs and 80 patients with SLE (all sporadic) from Cohort #2 (right panel). Bars denote means of variant frequency. ***P<0.0001 (two-tailed Student’s t-test). (C) Sanger-sequencing chromatograms for heterozygous variants at GLK (A410T), (K650R) and 3′-UTR (A644C). Arrows indicate the bases with distinct nucleotides. Y denotes mixed bases of C and T nucleotides; K denotes mixed bases of G and T nucleotides. HCs, healthy controls; PBMCs, peripheral blood mononuclear cells; SLE, systemic lupus erythematosus; UTR, untranslated region.

To identify GLK gene variants in patients with SLE, we isolated genomic DNAs of peripheral blood mononuclear cells (PBMCs) from 101 patients with SLE and 163 individuals without SLE (6 healthy controls (HCs) and 157 family members of patients with SLE) (Cohort #1, Taichung Veterans General Hospital, located in Taichung City in central Taiwan; online supplemental table S1). The genomic DNAs were subjected to next-generation sequencing for GLK exons and the 3′-untranslated region (UTR) with the sequencing depth of around 100 000 reads. One GLK somatic variant 3′-UTR (T635C), hg19 human reference genome chr2:39 477 124 A>G, was identified in patients with SLE and individuals without SLE (HCs and family members without SLE) with variant frequencies of 0%–5.3% and 0%–2.3%, respectively (figure 1A and B, left panels). The means of variant frequencies of SLE versus groups without SLE were not significantly different due to high SD (1.47%) of the variant frequency in patients with SLE. Interestingly, several patients with SLE showed higher frequency of this GLK somatic variant 3′-UTR (T635C) compared with the group without SLE (figure 1B, left panel and online supplemental figure S1). To investigate the potential significance of these frequency values, we determined the cut-off value of the somatic mutation frequencies between patients with SLE and individuals without SLE by using the values of mean plus 3SD of individuals without SLE (2.7%, 99.7% of normal distribution) according to Westgard rules. Seventeen (16.83%) of 101 patients with SLE (or 10 (12.99%) of 77 family members without SLE), but no HCs nor family members without SLE, showed a variant frequency of 2.7% or higher (figure 1B, left panel; online supplemental figure S1 and table S2). Next, we studied whether there is a potential association between the high frequency of the GLK somatic variant 3′-UTR (T635C) and SLE. We found that higher frequencies (>2.7%) of GLK somatic variant 3′-UTR (T635C) were associated with SLE in Cohort #1 (p<0.0001; table 1). Additional four GLK somatic missense variants were also identified in other Cohort #1 patients with SLE but not in individuals without SLE (table 1). Moreover, a GLK germline variant (50.6% read frequency) 3′-UTR (A644C) (chr2:39,477,115 T>G) was also identified in another female patient with SLE of Cohort #1 (0. 581% allele frequency; table 2); this variant is a previously annotated single nucleotide polymorphism (SNP), rs191224999. It is noted that one male family member without SLE of this patients with SLE also harboured this germline variant (table 2). In addition to GLK 3′-UTR, three (all females) of 101 Cohort #1 patients with SLE showed a GLK germline variant at the codon p.Ala410 to Thr (GCA to ACA) with 1.163% allele frequency (figure 1A and table 2); this variant is a previously annotated SNP, rs148167737. There are no Cohort #1 individuals without SLE harbouring this GLK (A410T) variant/SNP (table 2). Interestingly, two of the three Cohort #1 patients with SLE with GLK p.Ala410Thr variant belong to the same family F7 (table 2). In addition, a second GLK germline variant, p.Lys650Arg, was identified in other two female patients with SLE from Cohort #1 (0.158% allele frequency; figure 1A and table 2); this germline variant is also the same as another previously annotated SNP, rs200566214. The two patients with SLE with GLK p.Lys650Arg germline variant belong to the same family F26 (figure 1A and table 2), whereas their healthy brother did not have GLK p.Lys650Arg variant. There are no Cohort #1 individuals without SLE harbouring the GLK (K650R) variant. The three abovementioned GLK germline variants were further confirmed by Sanger sequencing (figure 1C).

10.1136/annrheumdis-2021-221010.supp1 Supplementary data

**Table 1:** GLK gene somatic variants resulting in codon or 3′-UTR changes in patients with SLE of Cohort #1 and Cohort #2

Cohort	Locus	Type	Ref	GLK coding	Codon/3′-UTR change	Patient number	Mutation frequency among reads	SLE ID#	Control number	Association with SLE
#1 (SLE, n=101) (control, n=163)	chr2:39 477 820	SNV	G	c.2624C>G	p.Thr875Ser	1	0.064	S5	0	p=0.383
	chr2:39 494 337	SNV	A	c.2025T>G	p.Cys675Trp	1	0.070		0	p=0.383
	chr2:39 507 491	SNV	C	c.1635G>A	p.Ala546Thr	2	0.044	S1	0	p=0.146
							0.076	S4		
	chr2:39 552 878	SNV	A	c.800T>A	p.Leu267Ter	1	0.033	S7	0	p=0.383
	chr2:39 477 124	SNV	A	c.3320T>C	3′-UTR U635C*	17	>0.027	S2, S7, S8, S16, S17, S29, S30, F10-4, F13-1, F14-1, F14-2, F15-1, F15-2, F18-1, F18-2, F19-3, F53-3	0	p<0.0001
#2 (SLE, n=80) (control, n=87)	chr2:39 499 454	INDEL	G	c.1942_1943 insert A	p.Ala648fs	1	0.016	B52	0	p=0.479
	chr2:39 553 291	SNV	T	c.658A>G	p.Met220Val	1	0.028		0	p=0.479
	chr2:39 553 305	SNV	A	c.644T>C	p.Phe215Ser	1	0.028		0	p=0.479
	chr2:39 553 354	SNV	C	c.595G>A	p.Ala199Thr	1	0.029		0	p=0.479
	chr2:39 492 369	SNV	G	c.2111C>A	p.Pro704Gln	1	0.026	B53	0	p=0.479
	chr2:39 499 497	SNV	C	c.1900G>T	p.Asp634Tyr	1	0.070		0	p=0.479
	chr2:39 517 440	SNV	G	c.1307C>T	p.Pro436Leu	1	0.031		0	p=0.479
	chr2:39 570 569	SNV	C	c.270G>A	p.Met90Ile	1	0.039		0	p=0.479
	chr2:39 583 402	SNV	C	c.233G>T	p.Gly78Val	1	0.026		0	p=0.479
	chr2:39 477 124	SNV	A	c.3320T>C	3′-UTR U635C^*^	37	>0.027	B14, B15, B16, B21, B22, B23, B24, B26, B29, B30, B31, B32, B37, B38, B39, B40, B41, B42, B47, B48, B53, B54, B55, B58, B61, B63, B64, B65, B67, B69, B70, B71, B73, B75, B76, B77, B79	14	p<0.0001

GLK coding, GLK variant coding that is a reverse sequence on chromosome 2.

Association of GLK somatic variants with SLE was determined by Fisher’s exact test (two-tailed).

‘F’ denotes family member in Cohort #1; ‘S’ denotes patient with sporadic SLE in Cohort #1; ‘B’ indicates patient with sporadic SLE in Cohort #2.

Ref, DNA coding from the human genome hg19 reference.

*Variant occurs in both Cohort #1 and Cohort #2.

fs, frameship; INDEL, insertion/deletion; SLE, systemic lupus erythematosus; SNV, single nucleotide variant; UTR, untranslated region.

**Table 2:** GLK gene germline variants* resulting in codon or 3′-UTR change in patients with SLE of Cohort #1 and Cohort #2

Locus	Ref	GLK coding	Codon/3′-UTR change	Annotated SNP	Cohort	Patient number	SLE ID#	Allele frequency in SLE	Allele frequency in control	Allele frequency in world
chr2:39 477 115	T	c.3329A>C	3′-UTR (A644C)	rs191224999	#1	1	F52-01	1/172 (0.005814)	1^†^/136 (0.007353)	0.000050
					#2	2	B24 B71	2/160 (0.012500)	0	
chr2:39 519 957	C	c.1228G>A	p.Ala410Thr	rs148167737	#1	3	S10, F7-01 F7-04	2/172 (0.01163)	0	0.000601
					#2	2	B33 B45	2/160 (0.012500)	1‡/174 (0.005747)	
chr2:39 499 448	T	c.1949A>G	p.Lys650Arg	rs200566214	#1	2	F26-01 F26-02	1/172 (0.001581)	0	0.000231
					#2	0	none	0	0	
chr2:39 505 607	C	c.1735G>A	p.Ala579Thr	ND	#2	1	B19	1/160 (0.006250)	0	ND

GLK coding, GLK variant coding that is a reverse sequence on chromosome 2.

Cohort #1, SLE, n=101 (24 patients with sporadic SLE and 77 patients with SLE from 62 families); non-SLE, n=163 (6 healthy controls and 157 family members without SLE from 62 families).

Cohort #2, SLE, n=80 (patients with sporadic SLE); healthy control, n=87 (non-familial healthy controls).

Allele frequencies were calculated using unrelated patients and controls; if from individual families, only one patient with SLE and one member without SLE from each family are included.

‘F’ denotes family member in Cohort #1; ‘S’ denotes patient with sporadic SLE in Cohort #1; ‘B’ indicates patient with sporadic SLE in Cohort #2.

*Single nucleotide variants.

†One male family member without SLE control (F52-02) from Cohort #1 harboured this variant.

‡One male non-familial healthy control from Cohort #2 harboured this variant.

Ref, DNA coding from the human genome hg19 reference; SLE, systemic lupus erythematosus; UTR, untranslated region.

To validate the abovementioned GLK gene variants in patients with SLE, we further recruited the second cohort containing 80 patients with sporadic SLE and 87 non-familial HCs from a different hospital (Kaohsiung Medical University Hospital; figure 1A, right panel and online supplemental table S3) located in another city, Kaohsiung City, in southern Taiwan. The genomic DNAs of PBMCs from Cohort #2 were subjected to next-generation sequencing with the sequencing depth of 100 000 to 3 00 000 reads. Consistently, the most prevalent GLK somatic variant in Cohort #1, GLK 3′-UTR (T635C), was also identified in Cohort #2 patients with SLE (table 1). The frequencies of this GLK somatic variant were also significantly increased in patients with SLE compared with those of HCs in Cohort #2 (figure 1B, right panel); 37 of 80 patients with SLE showed the variant frequency higher than 2.7%, whereas only 14 of 87 HCs did (figure 1B, right panel). Consistent with Cohort #1, GLK somatic variant 3′-UTR (T635C) with higher frequency (>2.7%) also showed a significant association with SLE in Cohort #2 (p<0.0001; table 1). Moreover, GLK 3′-UTR (A644C) germline variant, identified in one patient with SLE in Cohort #1, was also detected in two female patients with SLE in Cohort #2 (table 2). In addition, GLK p.Ala410Thr germline variant, identified in three patients with SLE in Cohort #1, was also detected in two female patients with SLE in Cohort #2 (table 2). It is noted that one male non-familial HC from Cohort #2 had GLK p.Ala410Thr germline variant (table 2). No additional patients with SLE from Cohort #2 showed any GLK p.Lys650Arg germline variant detected in two patients with SLE from Cohort #1 (table 2). Another GLK germline variant, p.Ala579Thr, was also detected in one female patient with SLE in Cohort #2 but not in Cohort #1 (table 2); this germline variant is not an annotated SNP. Furthermore, other two Cohort #2 patients with SLE each has four or five GLK somatic missense variants (table 1); these somatic variants were not detected in any Cohort #1 patients. Next, we studied whether the abovementioned GLK gene variants are involved in GLK dysregulation.

### Somatic and germline variants in the GLK 3′-UTR cause induction of GLK mRNA levels

**Figure 2:**
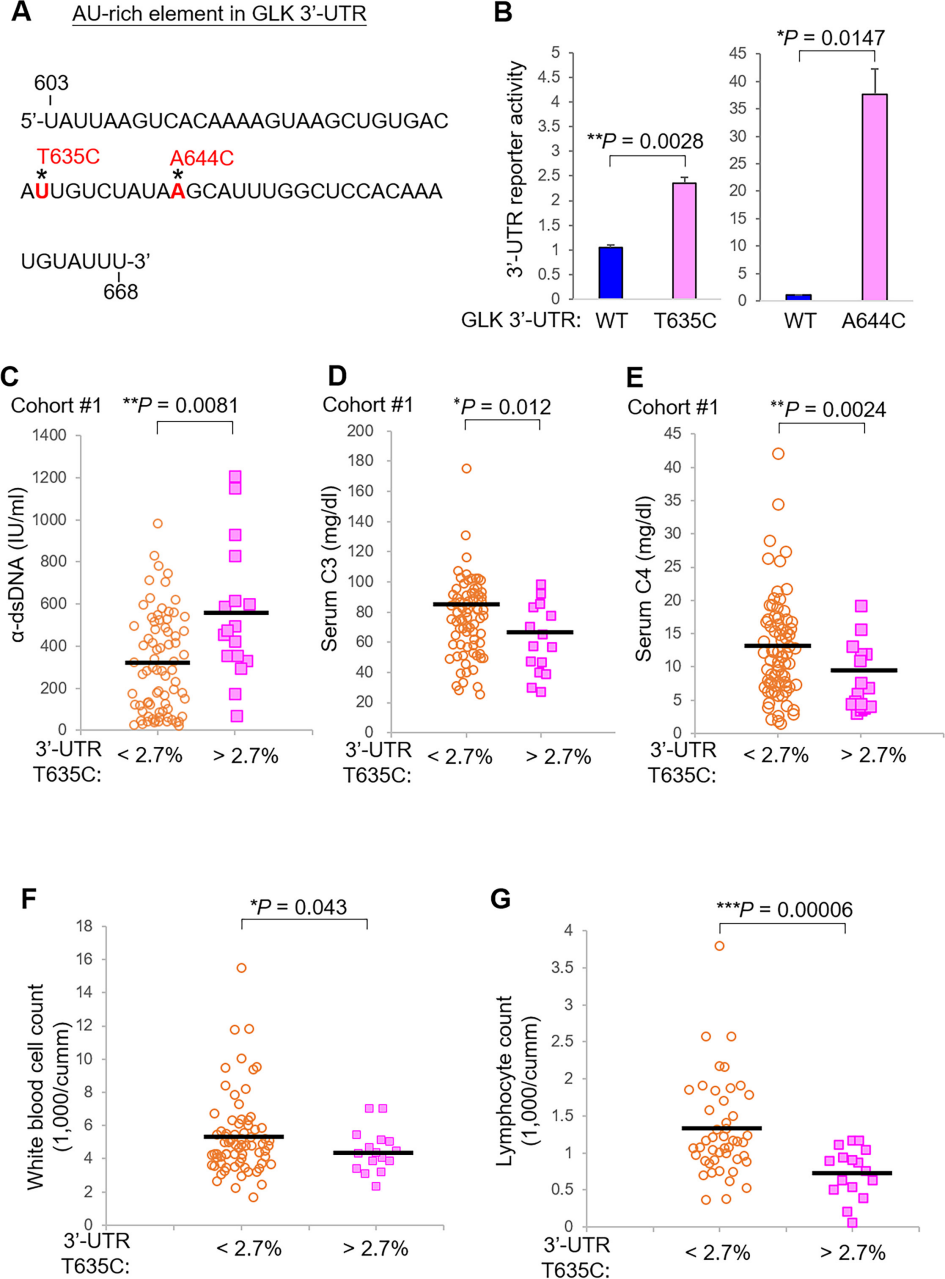
GLK 3′-UTR (T635C) variant results in GLK overexpression and is associated with severe symptoms from Cohort #1. (A) The AU-rich element (UTR nucleotide number: 603 to 668) in the GLK 3′-UTR. Asterisks indicate the location of GLK 3′-UTR (T635C) or (A644C) variant. (B) Bar charts of GLK 3′-UTR reporter activity in 3′-UTR wild-type, T635C or A644C mutant-expressing Jurkat T cells. The reporter activity of GLK 3′-UTR-gaussia luciferase (Luc) was normalised to the secreted alkaline phosphatase. Means±SEM are shown. (C) Anti-double-stranded DNA antibody (α-dsDNA) levels in the sera of Cohort #1 patients with SLE with a lower (<2.7%, n=77) or higher (>2.7%, n=17) variant frequency. (D) Serum C3 levels of Cohort #1 patients with SLE with a lower (<2.7%, n=77) or higher (>2.7%, n=17) variant frequency. (E) Serum C4 levels of Cohort #1 patients with SLE with a lower (<2.7%, n=76) or higher (>2.7%, n=17) variant frequency. (F) White blood cell count in the peripheral blood of Cohort #1 patients with SLE with a lower (<2.7%, n=74) or higher (>2.7%, n=16) variant frequency. (G) Lymphocyte count in the peripheral blood of Cohort #1 patients with SLE with a lower (<2.7%, n=58) or higher (>2.7%, n=16) variant frequency. Bars denote means of levels. *P <0.05; **p<0.01; ***p<0.001 (two-tailed Student’s t-test). SLE, systemic lupus erythematosus; UTR, untranslated region.

AU-rich elements (AREs) in the 3′ UTR induce mRNA destabilisation.^19 20^ GLK 3′-UTR (T635C) somatic variant and (A644C) germline variant were in a putative ARE, which contained 62 nucleotides with 69.4% AU nucleotides (figure 2A); therefore, we studied whether GLK 3′-UTR (T635C) somatic variant or (A644C) germline variant affects GLK mRNA levels using luciferase (Luc) reporter assays. The reporter activity of GLK 3′-UTR (T635C)-Luc was significantly increased (2.35 times) compared with that of wild-type GLK 3′-UTR-Luc (figure 2B, left panel). Besides GLK 3′-UTR (T635C) mutation, GLK 3′-UTR (A644C) mutation also drastically enhanced the reporter activity (figure 2B, right panel). These results suggest that the GLK 3′-UTR (T635C) or (A644C) variant increases GLK mRNA levels. Consistently, analysis of T cells from a cohort reported previously^13^ also showed that GLK mRNA levels were increased in 84.6% (11 of 13) of patients with SLE compared with those of HCs (online supplemental figure S2).

### GLK 3′-UTR (T635C) somatic variant is associated with increased anti-dsDNA and decreased serum C3/C4 levels

**Figure 3:**
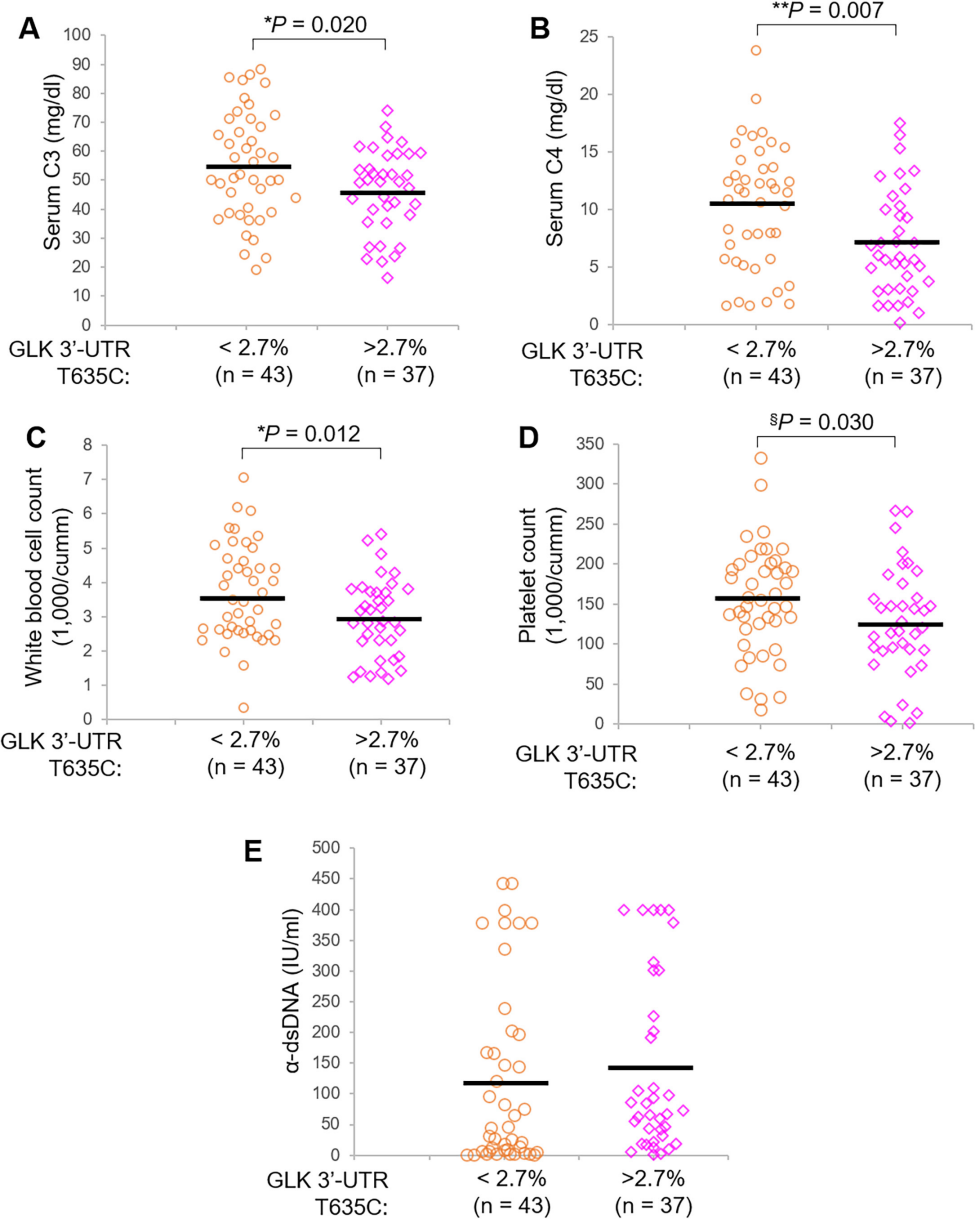
GLK 3′-UTR (T635C) variant is also associated with severe symptoms of patients with SLE from Cohort #2. Complement C3 levels (A) and complement C4 levels (B) in the sera of Cohort #2 patients with SLE with a lower (<2.7%, n=43) or higher (>2.7%, n=37) mutation frequency. White blood cell count (C) and platelet count (D) in the peripheral blood of Cohort #2 patients with SLE with a lower (<2.7%, n=43) or higher (>2.7%, n=37) mutation frequency. (E) Anti-double stranded DNA antibody (α-dsDNA) levels in the sera of Cohort #2 patients with SLE with a lower (<2.7%, n=43) or higher (>2.7%, n=37) mutation frequency. Bars denote means of levels. *P<0.05; **p<0.01 (two-tailed student’s t-test); ^§^p<0.05 (one-tailed Student’s t-test). SLE, systemic lupus erythematosus; UTR, untranslated region.

To study the clinical consequences of the GLK 3′-UTR (T635C) somatic variant, we analysed clinical parameters of patients with SLE from Cohort #1. Patients with SLE with higher variant frequency (>2.7%) of GLK 3′-UTR (T635C) showed an induction of anti-dsDNA autoantibody levels compared with those of patients with SLE with lower variant frequency (<2.7%) (figure 2C). Consistently, the patients with SLE with higher variant frequency also showed decreased serum complement C3 and C4 levels during the follow-up period (figure 2D and E); these patients also showed decreased cell counts of white blood cells (WBCs) and lymphocytes in the peripheral bloods (figure 2F and G). Interestingly, the patients with SLE with higher variant frequency (>2.7%) of GLK 3′-UTR (T635C) somatic variant showed a higher mean value of SLE disease activity index (SLEDAI), although statistically insignificant, compared with that of patients with SLE with lower variant frequency (<2.7%) (figure 3A). Furthermore, higher frequencies (>2.7%) of GLK 3′-UTR (T635C) somatic variant in Cohort #1 were associated (p=0.057) with higher scores of SLEDAI (online supplemental table S4). It is plausible that the association between GLK 3′-UTR (T635C) somatic variant and SLEDAI may achieve statistical significance after enrolling more patient samples. In addition, higher frequencies (>2.7%) of GLK 3′-UTR (T635C) somatic variant in Cohort #1 were not associated with the development of rashes, oral ulcer, arthritis, serositis, neuropsychiatric, nephritis, as well as the treatment with cyclophosphamide (Endoxan), mycophenolate mofetil, cyclosporine or azathioprine (online supplemental table S4).

Consistent with the data derived from Cohort #1, Cohort #2 patients with SLE with a higher variant frequency (>2.7%) of GLK 3′-UTR (T635C) also showed significantly decreased serum C3/C4 levels, WBC counts and platelet counts (figure 3A–D). These patients in Cohort #2 showed an increased mean value of anti-dsDNA levels but without statistical significance (figure 3E); this may be due to a smaller patient number of Cohort #2 than that of Cohort #1. Similar to the results of Cohort #1, the GLK 3′-UTR (T635C) somatic variant in Cohort #2 was also not associated with any organ damage or therapeutic treatment (online supplemental figure S3B and table S5). The data suggest that patients with SLE with the GLK 3′-UTR (T635C) somatic variant could develop severe SLE symptoms such as inflammation and lymphocytopaenia.

### GLK germline variants A410T and K650R elicit GLK protein stabilisation

**Figure 4:**
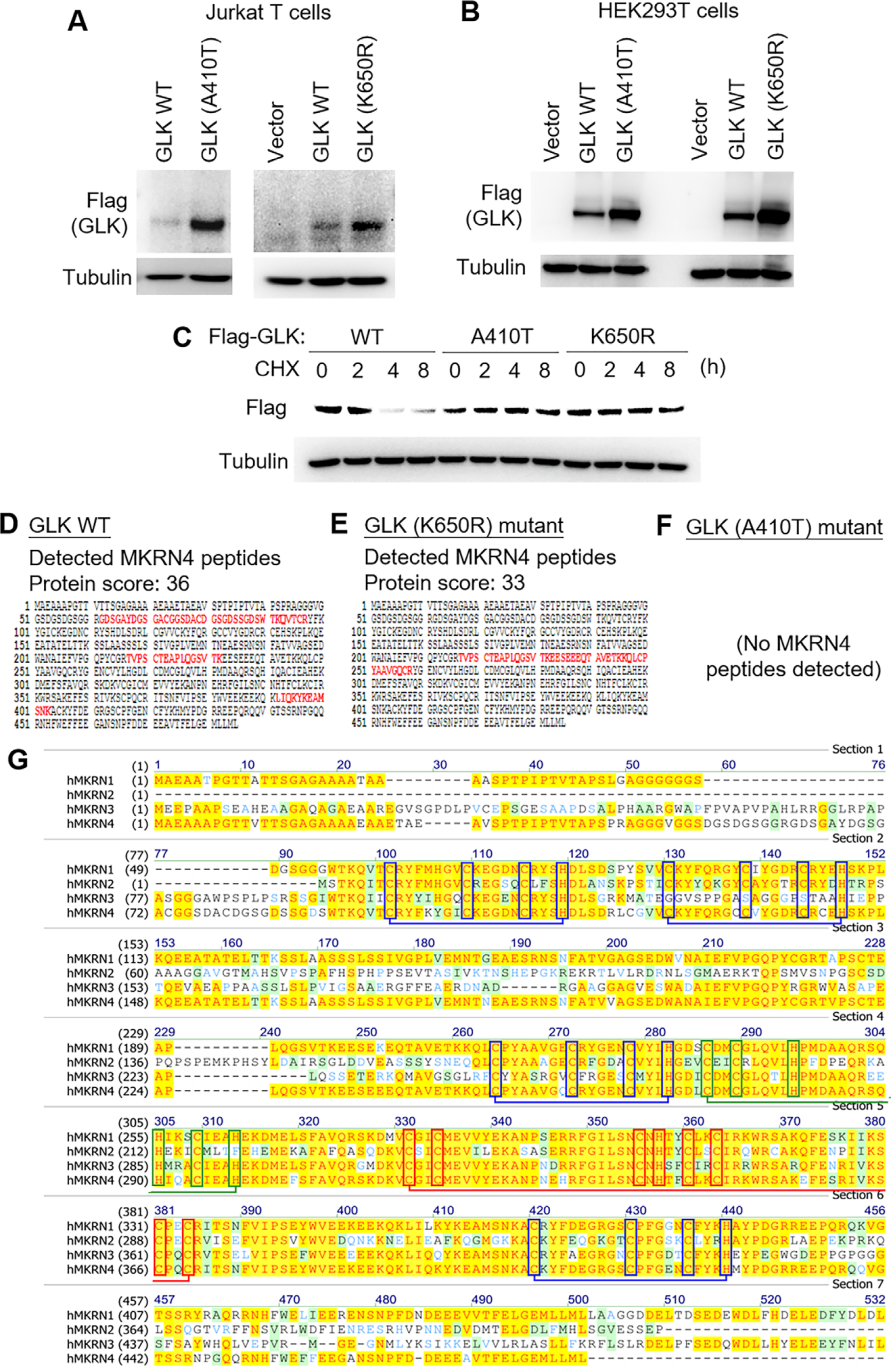
GLK (A410T) or GLK (K650R) variant enhances GLK protein stability. (A) Immunoblotting of Flag-tagged GLK and tubulin proteins from Jurkat T cells transfected with Flag-GLK WT), A410T mutant, or K650R mutant plasmid. Transfected Jurkat T cells were harvested at 48 hours post-transfection, followed by immunoblotting analyses. (B) Immunoblotting of Flag-tagged GLK and tubulin proteins from HEK293T cells transfected with Flag-GLK WT, A410T mutant or K650R mutant plasmid. Transfected HEK293T cells were harvested at 12 hours post-transfection, followed by immunoblotting analyses. (C) Cycloheximide pulse-chase experiments using HEK293T cells. immunoblotting of Flag-tagged GLK (anti-FLAG) and tubulin proteins from HEK293T cells transfected with Flag-GLK WT), A410T mutant or K650R mutant plasmid. Transfected cells were treated with 100 µg/mL CHX for up to 24 hours. (D, E) The detected peptide sequences (red colour) of the endogenous MKRN4 proteins by mass spectrometry analyses using the Flag-tagged GLK immunocomplex isolated from the Jurkat T cells that were transfected with either Flag-GLK WT or Flag-GLK (K650R) mutant plasmid. (F) No MKRN4 peptides detected using the Flag-tagged GLK (A410T)-immunocomplex. The immunocomplex was isolated from the Jurkat T cells that were transfected with Flag-GLK (A410T) plasmid. (G) Protein sequence alignment of human MKRN1, MKRN2, MKRN3 and MKRN4. Amino acids highlighted in yellow and dark green represent the conserved and the similar amino acids, respectively. Amino acids shown in light green represent the weakly similar amino acids. Red, blue and green boxes denote C3HC4 ring domain, C3H domain and MKRN motif, respectively. CHX, cycloheximide; WT, wild-type.

Five patients with SLE (three in Cohort #1 and two in Cohort #2) harboured GLK (A410T) germline variant (table 2). Two other patients with SLE from one family in Cohort #1 harboured GLK (K650R) germline variant (table 2). One patient with SLE (#S5) in Cohort #1 harboured two GLK (T875S and C675W) somatic variants (table 1). In Cohort #2, GLK (A579T) germline variant was identified from one patient with SLE (table 2). Two non-familial patients with SLE (#B52 and #B53) in Cohort #2 harboured multiple somatic variants (table 1). To study the functional consequence of these GLK variants that altered GLK codons, we performed mutagenesis and immunoblotting analyses. The protein levels of GLK (A410T) and GLK (K650R) mutants were increased compared with those of wild-type GLK in transfected Jurkat T cells (figure 4A) and HEK293T cells (figure 4B), whereas protein levels of GLK (C675W) and GLK (T875S) mutants were modestly increased (online supplemental figure S4A). Moreover, GLK levels were also increased by GLK (A579T) germline variant identified from one Cohort #2 patient, as well as GLK (A199T), GLK (A648fs), GLK (G78V), GLK (M90I), GLK (P436L) and GLK (D634Y) somatic variants identified from two Cohort #2 non-familial patients (#B52 and #B53) (online supplemental figure S4B).

**Figure 5:**
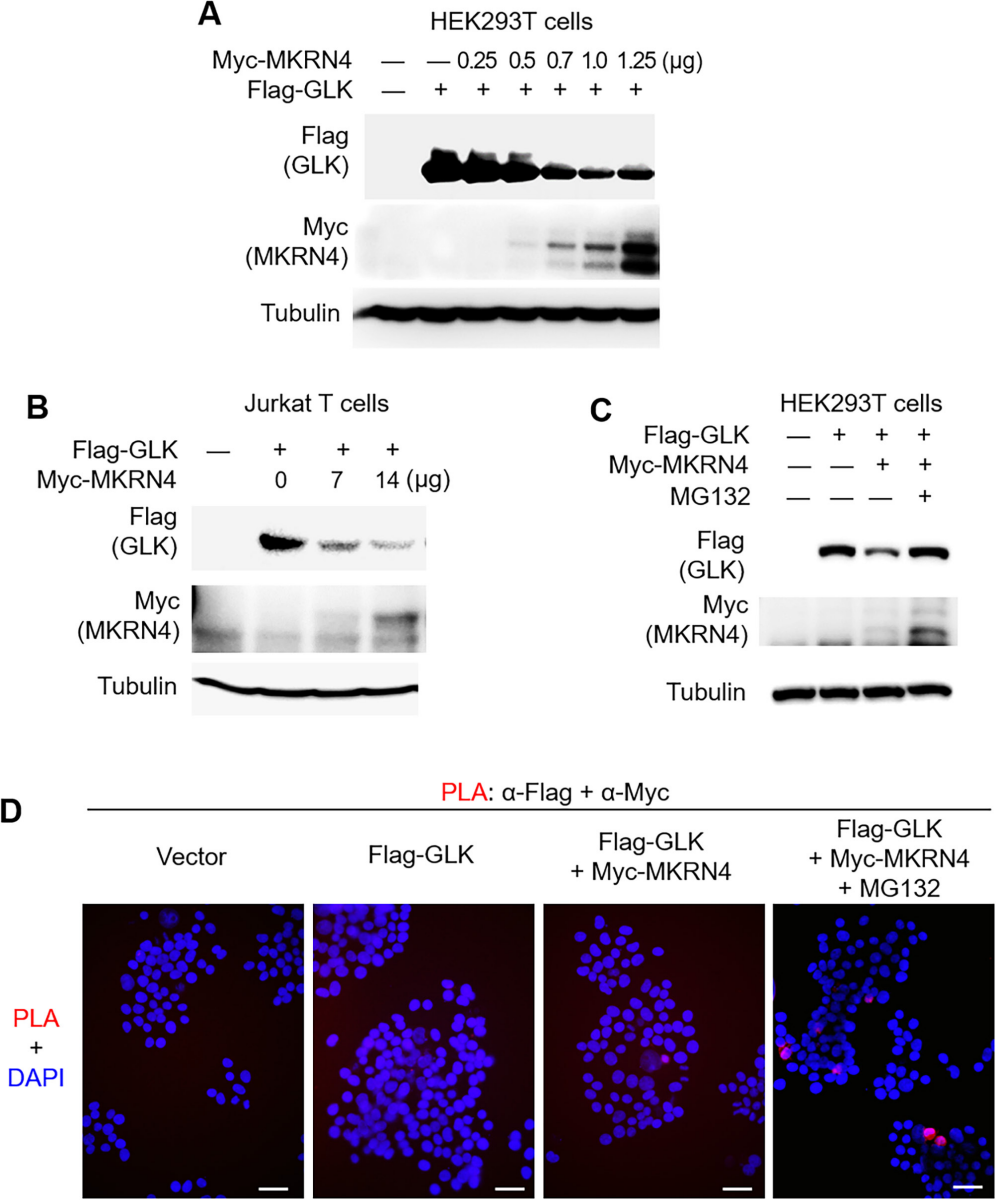
The novel E3 ligase MKRN4 induces proteasomal degradation of GLK. (A) Immunoblotting of Flag-tagged GLK (anti-FLAG), Myc-tagged MKRN4 (anti-MYC) and tubulin proteins from Jurkat T cells cotransfected with Flag-GLK plus increasing amounts of Myc-MKRN4 plasmids. (B) Immunoblotting of Flag-tagged GLK (anti-FLAG), Myc-tagged MKRN4 (anti-MYC) and tubulin proteins from HEK293T cells cotransfected with Flag-GLK plus increasing amounts of Myc-MKRN4 plasmids. (C) Immunoblotting of Flag-tagged GLK (anti-FLAG), Myc-tagged MKRN4 (anti-MYC) and tubulin proteins from HEK293T cells cotransfected with Flag-GLK plus Myc-MKRN4 plasmids. Cells were treated with 25 µM MG132 for 2 hours before being harvested. (D) In situ PLA assays of the interaction between Myc-tagged MKRN4 and Flag-tagged GLK proteins in HEK293T cells. Cells were treated with 25 µM MG132 for 2 hours before being harvested. Nuclei were stained with 4′,6-diamidino-2-phenylindole (DAPI). Imaging was detected by Leica DM2500 upright fluorescence microscope. Original magnification, ×200. scale bars, 50 µm. PLA, proximity ligation assay.

GLK (A410T) and GLK (K650R) variants were the two most prevalent germline variants in both Cohort #1 and #2; thus, we further investigated the mechanism of GLK protein induction by GLK (A410T) and GLK (K650R) variants. To study whether GLK (A410T) or GLK (K650R) variant enhances its protein stability, the protein half-life of GLK was determined by cycloheximide pulse-chase experiments. GLK (A410T) and GLK (K650R) mutants showed longer GLK protein half-life in HEK293T cells (figure 4C), suggesting that GLK (A410T) and GLK (K650R) mutants are resistant to protein degradation. To identify the protease that targets and degrades GLK proteins, individual immunocomplexes of wild-type GLK, GLK (A410T) mutant and GLK (K650R) mutant were subjected to mass spectrometry-based proteomics analyses. The mass data revealed a novel E3 ligase, MKRN4, as an interacting protein of wild-type GLK or GLK (K650R) mutant but not GLK (A410T) mutant (figure 4D–F). To date, there are no known functions of MKRN4, a putative ubiquitin E3 ligase of MKRN family. After close examination of MKRN4 protein sequence, we found that MKRN4 did not show any deficiency in MKRN family conserved domains (figure 4G). Thus, we tested whether MKRN4 induces GLK protein degradation. Remarkably, MKRN4 overexpression induced GLK protein degradation in HEK293T cells and Jurkat T cells (figure 5A and B). MKRN4-induced GLK degradation was blocked by the proteasome inhibitor MG132 (figure 5C). In addition, the protein–protein interaction between GLK and MKRN4 was confirmed by in situ proximity ligation assays (figure 5D).

### GLK (A410T) and GLK (K650R) variants block MKRN4-induced Lys48-linked ubiquitination of GLK

**Figure 6:**
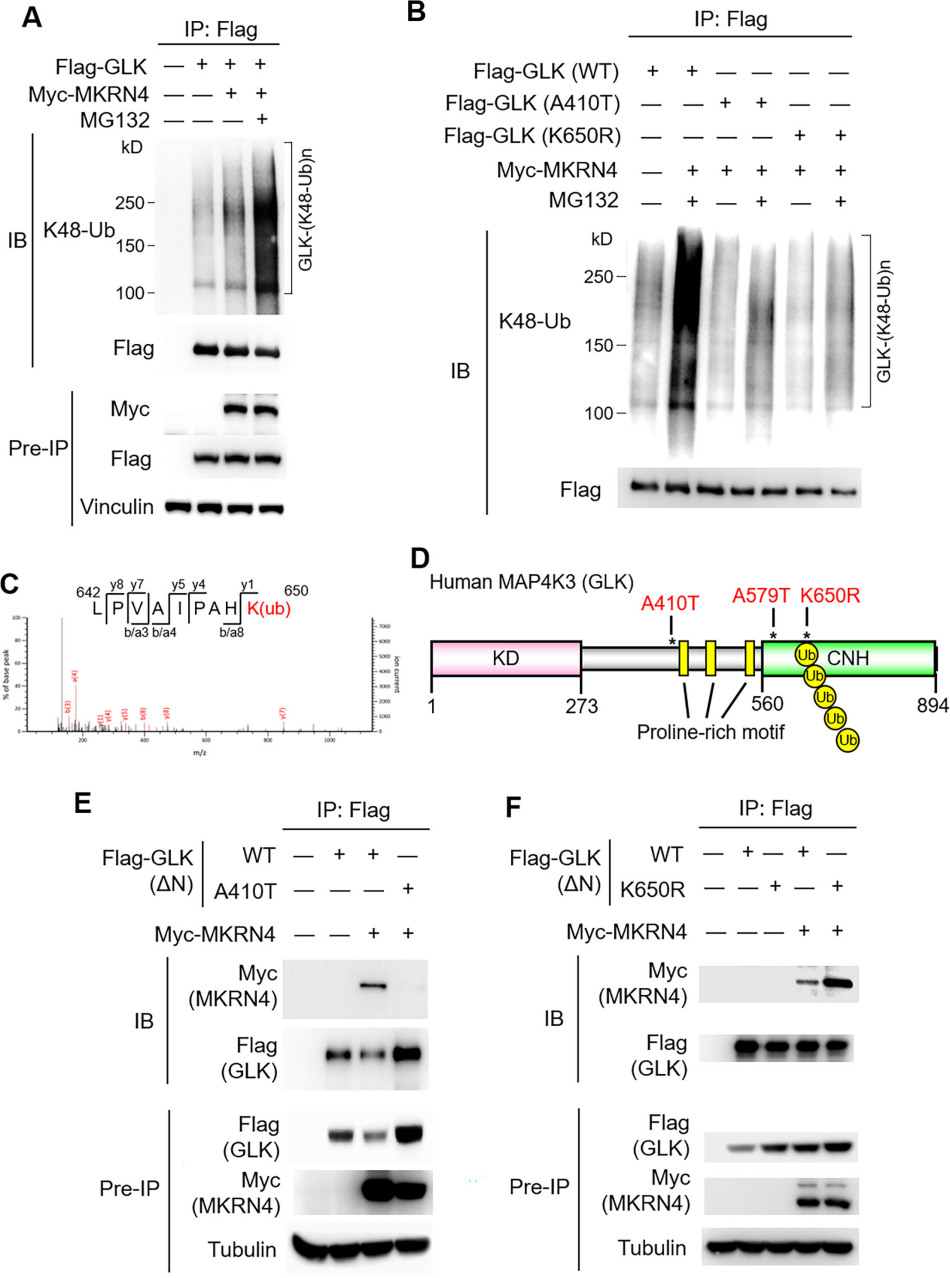
GLK (A410T) or GLK (K650R) mutant is resistant to MKRN4-induced GLK ubiquitination. (A) MKRN4-induced GLK ubiquitination. Flag-tagged GLK proteins were immunoprecipitated from lysates of HEK293T cells cotransfected with Flag-GLK plus Myc-MKRN4 plasmids, followed by immunoblotting with anti-Lys48-linked ubiquitination or anti-FLAG antibody. Cells were treated with 25 µM MG132 for 2 hours before being harvested. (B) Reduced GLK ubiquitination by GLK (A410T) or GLK (K650R) variant. Flag-tagged GLK proteins were immunoprecipitated from lysates of HEK293T cells cotransfected with Myc-MKRN4 plus Flag-GLK WT), A410T mutant, or K650R mutant, followed by immunoblotting with anti-Lys48-linked ubiquitination or anti-FLAG antibody. Cells were treated with 25 µM MG132 for 2 hours before being harvested. (C) Mass spectrometry analysis of the GLK peptides from the MKRN4 immunocomplex. The GLK peptide sequences containing Ub-Lys650 residue of GLK proteins that were detected in the MKRN4 immunocomplex are shown. (D) The structural domains of human MAP4K3 (GLK). Asterisks indicate the locations of A410T, K650R and A579T variants on GLK. (E) Coimmunoprecipitation of Flag-tagged GLKΔN with Myc-tagged MKRN4 proteins from lysates of HEK293T cells cotransfected with Myc-MKRN4 plus either Flag-GLKΔN (deletion of amino acids 1–272) wild-type or Flag-GLKΔN (A410T) mutant plasmids. (F) Coimmunoprecipitation of Flag-tagged GLK with Myc-tagged MKRN4 proteins from lysates of HEK293T cells transfected with Myc-MKRN4 plus either Flag-GLKΔN wild-type or Flag-GLKΔN (K650R) mutant plasmids. CNH, citron-homology domain; KD, kinase domain; WT, wild-type.

To investigate the molecular mechanism of GLK protein stabilisation by A410T or K650R mutation, we first tested whether MKRN4 induces the ubiquitination and subsequent proteasomal degradation of GLK. Immunoprecipitation and immunoblotting analyses showed that MKRN4 overexpression induced Lys48-linked ubiquitination of GLK (figure 6A), and the MKRN4-induced GLK ubiquitination was further enhanced by MG-132 treatment (figure 6A). Conversely, Lys48-linked ubiquitination of GLK was blocked by A410T or K650R mutation of GLK (figure 6B). To identify MKRN4-induced ubiquitination residues on GLK, MKRN4 immunocomplex was subjected to mass spectrometry-based analyses (online supplemental figure S5A). Interestingly, Lys650 residue of GLK proteins in the MKRN4 immunocomplex was identified as a MKRN4-targeted GLK ubiquitination site (figure 6C). Besides Lys650 residue, three additional lysine residues (Lys526, Lys550 and Lys620) were also identified as MKRN4-induced GLK ubiquitination sites (online supplemental figure S5A). Individual mutations of these three lysine residues did not block the MKRN4-induced K48-linked ubiquitination of GLK (online supplemental figure S5B). Conceivably, GLK (K650R) mutation would block MKRN4-induced ubiquitination of Lys650 residue on GLK, leading to GLK protein stabilisation. In addition, GLK (A410T) mutation is in the GLK proline-rich domain (figure 6D), which mediates protein–protein interaction.^21 22^ We next studied whether the interaction between MKRN4 and GLK is attenuated by GLK (A410T) mutation. To avoid the false-positive result due to the kinase-domain-mediated dimerisation between GLK (A410T) mutant and the endogenous wild-type GLK, GLK proteins without the GLK kinase domain (GLKΔN) were used. Coimmunoprecipitation analysis showed that the interaction between MKRN4 and wild-type GLKΔN was abolished by GLK (A410T) mutation (figure 6E). This result was consistent with our proteomics data that no MKRN4 peptides were detected in GLK (A410T) immunocomplex (figure 4F). Interestingly, GLK (K650R) mutation did not attenuate the GLK–MKRN4 interaction (figure 6F). This result suggests that GLK (A410T) mutation blocks its interaction with MKRN4, leading to GLK protein stabilisation. Collectively, either A410T or K650R mutation stabilises GLK proteins by preventing MKRN4-mediated ubiquitination and subsequent proteasomal degradation.

Our data showed that both GLK somatic and germline variants in patients with SLE lead to increased GLK levels. Induction of GLK in T cells contributes to IL-17A production and subsequent autoimmune responses.^14 17^ Thus, we studied whether the identified GLK variants are correlated with IL-17A induction in patients with SLE. The serum IL-17A levels were significantly increased in patients with SLE who harboured GLK germline or somatic variants compared with those of HCs in Cohort #1 (online supplemental figure S6), while IL-17A levels were modestly increased in patients with SLE without GLK variants (online supplemental figure S6). It is interesting that two patients with SLE (#S9 and #S12) without GLK variants showed high levels of serum IL-17A, which could be due to dysregulation or mutation of MKRN4. Collectively, these results suggest that GLK variants contribute to induction of GLK levels and overproduction of IL-17A, leading to autoimmune responses.

## Discussion

A key finding of this study was the identification of one recurrent somatic variant (3′-UTR (T635C)) and four germline variants (3′-UTR (A644C), A410T, A579T or K650R) of GLK in a subgroup of patients with SLE from two independent cohorts. These variants cause GLK overexpression. GLK overexpressing and GLK^+^ IL-17A^+^ T-cell subpopulations are correlated with SLE disease activity of human patients with SLE.^17^ Previous reports demonstrate that GLK overexpression in T cells induces IL-17A overproduction, leading to autoimmune responses^14^; conversely, GLK inhibitor blocks IL-17A production from human SLE T cells and attenuates disease severity of autoimmune disease mice.^17^ The findings reported here suggest that the 3′-UTR (T635C), 3′-UTR (A644C), A410T, A579T or K650R variant-induced GLK overexpression through the stabilisation of GLK mRNAs or proteins may contribute to SLE pathogenesis.

One of the exciting findings in this report is the identification of the novel E3 ligase MKRN4 that induces GLK protein degradation. This is the first report revealing that MKRN4 is an E3 ubiquitin ligase instead of a pseudogene. MKRN4 ubiquitinated GLK at Lys650 residue. Consistently, GLK (K650R) mutation blocked MKRN4-induced Lys48-linked ubiquitination of GLK; GLK (A410T) mutation attenuated its association with MKRN4. Interestingly, GLK (A648fs) somatic frameshift variant results in the lack of Lys650 residue―the MKRN4-targetted GLK ubiquitination site; therefore, GLK (A648fs) mutation causes GLK protein induction by preventing MKRN4-induced protein degradation. Thus, A410T, K650R or A648fs variant of GLK causes GLK protein stabilisation by blocking MKRN4-mediated GLK ubiquitination.

SLE is a multigenic disease associated with genetic, environmental and gender factors.^23 24^ Patients with SLE with GLK 3′-UTR (T635C) somatic variant showed more severe inflammation (increased anti-dsDNA antibody and decreased C3/C4 levels) and lymphocytopaenia than those of patients with SLE without this somatic variant. T-cell-specific GLK transgenic mice display high levels of autoantibodies and severe inflammation.^14^ Thus, the severe SLE symptoms may be due to GLK overexpression induced by T635C variant, as well as other somatic or germline variants of GLK. GLK 3′-UTR (T635C) somatic variant (>2.7% frequency) occurs in 17 (16.8%) of 101 patients with SLE from Cohort #1. Notably, none of any HCs nor family members without SLE from all 62 families in Cohort #1 showed high frequency of this somatic variant. These results suggest that the GLK 3′-UTR (T635C) somatic variant in Cohort #1 is not inherited and is independent of their family environment. In Cohort #2, 37 (46.3%) of 80 patients with SLE and 14 (16.1%) of 87 HCs harboured GLK 3′-UTR (T635C) somatic variant (>2.7% frequency); the numbers of patients with SLE and HCs who harboured GLK 3′-UTR (T635C) somatic variant in Cohort #2 were higher than those of Cohort #1. Interestingly, the age of SLE in Cohort #2 (median: 43.5 years old) was older than that of Cohort #1 (median: 31 years old). Somatic mutation accumulation is associated with ageing due to the increase of clonal haematopoiesis^25 26^; therefore, the increased frequencies of GLK 3′-UTR (T635C) in Cohort #2 patients with SLE (with older age than Cohort #1) may be due to clonal haematopoiesis increasing with age. However, median age of the control group was not significantly different between Cohort #1 (median: 40 years old) and Cohort #2 (median: 42 years old); the Cohort #2 HC group showed a slightly higher mean value of GLK 3′-UTR (T635C) somatic variant frequency than that of Cohort #1. Notably, Cohort #2 individuals were enrolled from the heavy industrial city Kaohsiung in southern Taiwan.^27^ Besides age, it is also possible that environmental pollutants may induce somatic variants on GLK, which could be one of the risk factors for SLE. Consistently, two patients with SLE (#B52 and #B53) from Cohort #2 had multiple somatic variants resulting in GLK codon changes and GLK protein induction. The findings suggest that individuals harbouring GLK variants accompanied by other risk factors could be at high risk for SLE.

GLK (A410T), (K650R) and 3′-UTR (A644C) variants are previously annotated, germline-transmitted SNPs. Besides these three SNPs, GLK (A579T) variant is a newly identified germline variant. Due to germline transmission, family members without SLE of the patients with SLE with these four GLK germline variants may also have these variants. Consistently, we noted that one male family member without SLE of a female patient with SLE also harboured GLK 3′-UTR (A644C) variant (table 1). The data suggest that complex risk factors, such as gender factors, in combination with GLK variant contribute to SLE pathogenesis. Two patients with SLE with GLK (K650R) variant were female siblings, whereas their healthy brother did not harbour GLK (K650R) variant. Due to the lack of DNA samples from other family members of patients, it is unclear whether GLK (A410T) variant, (K650R) variant or (A579T) variant occurs in other family members. Nevertheless, our findings suggest that individuals or family members with these four identified GLK germline variants may need to be vigilant for SLE or other autoimmune diseases. In addition, GLK (A410T) and (K650R) variants are the previously annotated SNP rs148167737 and SNP rs200566214, respectively; both SNPs are prevalent in Asia. The prevalence of GLK (A410T) variant/SNP in the world population is 0.000601, whereas it is 0.0017 in Asia, 0.0013 in East Asia and 0.0027 in other Asian (Asian individuals excluding South or East Asian) regions.^28^ The prevalence of GLK (K650R) variant/SNP in the world population is 0.000231, while it is 0.0036 in Asia, 0.0042 in East Asia and 0.0022 in other Asian regions.^28^ Notably, GLK 3′-UTR (A644C) variant, the SNP rs191224999, is barely identified in the world population (0.00005),^28^ but is frequently identified in Vietnamese population (0.014; NCBI BioProject number: 515199) and Korean population (0.0015).^29^ These three Asia-prevalent SNPs may be associated with the higher prevalence of SLE in Asia^30^ than that worldwide.

Multiple GLK somatic variants on the coding region (C675W, T875S, A579T, A199T, A648fs, G78V, M90I, P436L, D634Y and P704Q) also caused GLK protein stabilisation; therefore, many GLK codons could be somatically mutated, leading to GLK protein induction and subsequent autoimmune responses. At least one third of patients with SLE show a high frequency of GLK overexpressing T cells,^13^ while 39% (71 of 181) of patients with SLE have (A410T) germline variant/SNP, (K650R) germline variant/SNP, (A579T) germline variant, 3′-UTR (A644C) germline variant or 3′-UTR (T635C) somatic variant of GLK. Besides the aforementioned GLK variants in coding region and 3′-UTR, it is likely that, mutations or epigenetic changes on the GLK promoter region, as well as downregulation/mutation of MKRN4 may also lead to GLK overexpression and subsequent induction of autoimmune responses. Moreover, genomic analyses of GLK using other SLE cohorts in Western countries may provide additional insights about GLK overexpression-mediated SLE pathogenesis. Taken together, individuals who harbour the aforementioned GLK germline or somatic variants may be at high risk for SLE.

## Data Availability

Data are available upon reasonable request. The data supporting the findings of this study are documented within the paper and are available from the corresponding author upon request.

## References

[1] Kaul A , Gordon C , Crow MK , et al . Systemic lupus erythematosus. Nat Rev Dis Primers 2016;2:16039. 10.1038/nrdp.2016.39 27306639

[2] Ulff-Møller CJ , Simonsen J , Kyvik KO , et al . Family history of systemic lupus erythematosus and risk of autoimmune disease: nationwide cohort study in Denmark 1977-2013. Rheumatology 2017;56:957–64. 10.1093/rheumatology/kex005 28339674

[3] Kuo C-F , Grainge MJ , Valdes AM , et al . Familial aggregation of systemic lupus erythematosus and coaggregation of autoimmune diseases in affected families. JAMA Intern Med 2015;175:1518–26. 10.1001/jamainternmed.2015.3528 26193127

[4] Costenbader KH , Gay S , Alarcón-Riquelme ME , et al . Genes, epigenetic regulation and environmental factors: which is the most relevant in developing autoimmune diseases? Autoimmun Rev 2012;11:604–9. 10.1016/j.autrev.2011.10.022 22041580

[5] Olsen NJ , Karp DR , Autoantibodies KDR . Autoantibodies and SLE: the threshold for disease. Nat Rev Rheumatol 2014;10:181–6. 10.1038/nrrheum.2013.184 24296678

[6] Pan L , Lu M-P , Wang J-H , et al . Immunological pathogenesis and treatment of systemic lupus erythematosus. World J Pediatr 2020;16:19–30. 10.1007/s12519-019-00229-3 30796732 PMC7040062

[7] Lou H , Wojciak-Stothard B , Ruseva MM , et al . Autoantibody-dependent amplification of inflammation in SLE. Cell Death Dis 2020;11:729. 10.1038/s41419-020-02928-6 32908129 PMC7481301

[8] Maddur MS , Miossec P , Kaveri SV , et al . Th17 cells: biology, pathogenesis of autoimmune and inflammatory diseases, and therapeutic strategies. Am J Pathol 2012;181:8–18. 10.1016/j.ajpath.2012.03.044 22640807

[9] Böhne A , Darras A , D'Cotta H , et al . The vertebrate makorin ubiquitin ligase gene family has been shaped by large-scale duplication and retroposition from an ancestral gonad-specific, maternal-effect gene. BMC Genomics 2010;11:721. 10.1186/1471-2164-11-721 21172006 PMC3022923

[10] Kaneko S , Aki I , Tsuda K , et al . Origin and evolution of processed pseudogenes that stabilize functional makorin1 mRNAs in mice, primates and other mammals. Genetics 2006;172:2421–9. 10.1534/genetics.105.052910 16415359 PMC1456392

[11] Gaudet P , Livstone MS , Lewis SE , et al . Phylogenetic-based propagation of functional annotations within the gene ontology consortium. Brief Bioinform 2011;12:449–62. 10.1093/bib/bbr042 21873635 PMC3178059

[12] Naulé L , Kaiser UB . Evolutionary conservation of MKRN3 and other makorins and their roles in puberty initiation and endocrine functions. Semin Reprod Med 2019;37:166–73. 10.1055/s-0039-3400965 31972861 PMC8603287

[13] Chuang H-C , Lan J-L , Chen D-Y , et al . The kinase GLK controls autoimmunity and NF-κB signaling by activating the kinase PKC-θ in T cells. Nat Immunol 2011;12:1113–8. 10.1038/ni.2121 21983831

[14] Chuang H-C , Tsai C-Y , Hsueh C-H , et al . GLK-IKKβ signaling induces dimerization and translocation of the AhR-RORγt complex in IL-17A induction and autoimmune disease. Sci Adv 2018;4:eaat5401. 10.1126/sciadv.aat5401 30214937 PMC6135549

[15] Chen D-Y , Chuang H-C , Lan J-L , et al . Germinal center kinase-like kinase (GLK/MAP4K3) expression is increased in adult-onset still's disease and may act as an activity marker. BMC Med 2012;10:84. 10.1186/1741-7015-10-84 22867055 PMC3424974

[16] Chen Y-M , Chuang H-C , Lin W-C , et al . Germinal center kinase-like kinase overexpression in T cells as a novel biomarker in rheumatoid arthritis. Arthritis Rheum 2013;65:2573–82. 10.1002/art.38067 23817999

[17] Chuang H-C , Chen Y-M , Chen M-H , et al . AhR-ROR-γt complex is a therapeutic target for MAP4K3/GLK^high^IL-17A^high^ subpopulation of systemic lupus erythematosus. Faseb J 2019;33:11469–80. 10.1096/fj.201900105RR 31318609 PMC6766655

[18] Chuang H-C , Tan T-H . MAP4K3/GLK in autoimmune disease, cancer and aging. J Biomed Sci 2019;26:82. 10.1186/s12929-019-0570-5 31640697 PMC6806545

[19] Chen CY , Shyu AB . Au-rich elements: characterization and importance in mRNA degradation. Trends Biochem Sci 1995;20:465–70. 10.1016/S0968-0004(00)89102-1 8578590

[20] Otsuka H , Fukao A , Funakami Y , et al . Emerging evidence of translational control by AU-rich element-binding proteins. Front Genet 2019;10:332. 10.3389/fgene.2019.00332 31118942 PMC6507484

[21] Chuang H-C , Wang X , Tan T-H . MAP4K family kinases in immunity and inflammation. Adv Immunol 2016;129:277–314. 10.1016/bs.ai.2015.09.006 26791862

[22] Diener K , Wang XS , Chen C , et al . Activation of the c-jun N-terminal kinase pathway by a novel protein kinase related to human germinal center kinase. Proc Natl Acad Sci U S A 1997;94:9687–92. 10.1073/pnas.94.18.9687 9275185 PMC23251

[23] Catalina MD , Owen KA , Labonte AC , et al . The pathogenesis of systemic lupus erythematosus: harnessing big data to understand the molecular basis of lupus. J Autoimmun 2020;110:102359. 10.1016/j.jaut.2019.102359 31806421

[24] Kwon Y-C , Chun S , Kim K , et al . Update on the genetics of systemic lupus erythematosus: genome-wide association studies and beyond. Cells 2019;8:1180. 10.3390/cells8101180 PMC6829439 31575058

[25] Dollé ME , Snyder WK , Gossen JA , et al . Distinct spectra of somatic mutations accumulated with age in mouse heart and small intestine. Proc Natl Acad Sci U S A 2000;97:8403–8. 10.1073/pnas.97.15.8403 10900004 PMC26960

[26] Xia J , Miller CA , Baty J , et al . Somatic mutations and clonal hematopoiesis in congenital neutropenia. Blood 2018;131:408–16. 10.1182/blood-2017-08-801985 29092827 PMC5790127

[27] Hsu C-Y , Chi K-H , Wu C-D , et al . Integrated analysis of source-specific risks for PM_2.5_-bound metals in urban, suburban, rural, and industrial areas. Environ Pollut 2021;275:116652. 10.1016/j.envpol.2021.116652 33588193

[28] Phan L , Jin Y , Zhang H . Alfa: allele frequency aggregator, 2020. National library of medicine. Available: https://www.ncbi.nlm.nih.gov/snp/docs/gsr/alfa/

[29] Jeon S , Bhak Y , Choi Y , et al . Korean genome project: 1094 Korean personal genomes with clinical information. Sci Adv 2020;6:eaaz7835. 10.1126/sciadv.aaz7835 32766443 PMC7385432

[30] Thumboo J , Wee HL . Systemic lupus erythematosus in Asia: is it more common and more severe? APLAR J Rheumatol 2006;9:320–6. 10.1111/j.1479-8077.2006.00235.x

