# Supplementary material for: Genomic sequencing and functional analyses identify MAP4K3/GLK germline and somatic variants associated with systemic lupus erythematosus
Source: Ann Rheum Dis. 2021 Oct 5;81(2):243–54. doi: 10.1136/annrheumdis-2021-221010 (PMC8762023; doi:10.1136/annrheumdis-2021-221010)
Supplement: Supplementary data [file annrheumdis-2021-221010supp001.pdf]

Online Supplemental Information

Genomic Sequencing and Functional Analyses Identify MAP4K3/GLK Germline and Somatic Variants Associated with Systemic Lupus Erythematosus

Table of contents

Materials and Methods	Pages 2-4
Supplemental Figure S1	Page 5
Supplemental Figure S2	Page 6
Supplemental Figure S3	Page 7
Supplemental Figure S4	Page 8
Supplemental Figure S5	Page 9
Supplemental Figure S6	Page 10
Supplemental Table S1	Page 11
Supplemental Table S2	Page 12
Supplemental Table S3	Page 13
Supplemental Table S4	Page 14
Supplemental Table S5	Page 15

Materials and Methods

Human samples

This study was conducted in accordance with the Helsinki Declaration. A total of 431 individuals, including 250 healthy/non-SLE individuals and 181 SLE patients were enrolled in this study (Cohort #1 and Cohort #2). No Taiwanese aborigines were enrolled in both cohorts. For Cohort #1, 163 non-SLE individuals (6 healthy controls and 157 non-SLE family members of SLE patients) and 101 SLE patients were referred to the Division of Immunology and Rheumatology at Taichung Veterans General Hospital in Taichung City, Taiwan. Briefly, 77 SLE patients and 157 non-SLE family members from 62 families were randomly enrolled to Cohort #1. Among these 62 families, 15 families each has two SLE patients. To enhance statistical power, 24 sporadic SLE patients and 6 healthy participants were further recruited to Cohort #1. All SLE patients fulfilled the 1997 ACR Revised Criteria for Classification of Systemic Lupus Erythematosus.[1] Disease activity was determined by SLEDAI score.[1]

For Cohort #2, 87 healthy individuals and 80 sporadic SLE patients were referred to the Division of Rheumatology at Kaohsiung Medical University Hospital in Kaohsiung City, Taiwan. Unlike Cohort #1, Cohort #2 did not recruit any individuals from the same family. All SLE patients fulfilled the 1982 Revised Criteria for Classification of Systemic Lupus Erythematosus.[2] Disease activity was determined by SLEDAI score.[2]

All study participants provided written informed consent. Peripheral blood collections from healthy controls and patients, as well as experiments were approved by the ethical committee of Taichung Veterans General Hospital (#C10130, #SE17193B) and of Kaohsiung Medical University Hospital (KMUHIRB-E(I)-20190108).

Next-generation sequencing (NGS)

Ten ng genomic DNAs from PBMCs were used for multiplex PCR of a panel covering the exon regions of the human map4k3 gene (Ion AmpliSeq Custom Panel, Life Technologies). Library construction of the amplicons and subsequent enrichment of the sequencing beads were performed using Ion Torrent Personal Genome Machine (Life Technologies) according to the manufacturer's protocol by Mission Biotech. DNA sequence data were analyzed by Variant Calling using built-in software Torrent Suite v5.10 and Partek Flow v10.0.21.0509. NGS using Ion Torrent system or Illumina system may have their respective weak points; nevertheless, the data derived from these two systems show a strong correlation (~0.93–0.97).[3]

Cell lines and transfection

The human Jurkat T leukemia cell line (ATCC, TIB-152) was cultured in RPMI-1640 medium; human embryonic kidney cell line (HEK293T; ATCC, CRL-11268) was cultured in DMEM medium. Both media were supplemented with 10% fetal bovine serum, 100 U/ml penicillin, and 100 mg/ml streptomycin (Gibco). All cells were free of mycoplasma contamination and grown at 37˚C in a humidified atmosphere of 5% CO_2 in air. Plasmids were transfected into HEK293T cells using polyethylenimine reagents (Sigma). Plasmids were transfected into Jurkat T cells by electroporation with the Neon Transfection System (Invitrogen) on 1,430 V for a duration of 30 ms and 1 pulse.

Reagents, antibodies, and plasmids

Cycloheximide was purchased from Sigma. Anti-Myc (clone 9E10), anti-Flag (clone M2), and anti-Lys48-linked ubiquitination (clone Apu2) monoclonal antibodies were purchased from Merck. Anti-Flag affinity gel (bead) and anti-Myc affinity gel (bead) were purchased from Merck. Rabbit anti-Flag polyclonal antibody (#ARG66332) was purchased from Arigo biolaboratories. Anti-tubulin (clone BT7R) monoclonal antibody was purchased from Thermo. Anti-GLK (clone C3) monoclonal antibody and Flag-GLK plasmid were reported previously.[4] The plasmid encoding

Chuang H-C, *et al. Ann Rheum Dis* 2022; 781:243–254. doi: 10.1136/annrheumdis-2021-221010

Flag-tagged human GLK ΔN was generated by subcloning the cDNA encoding GLK proline-rich motif and CNH domain (amino acids 273-894) into the vector pCMV6-AN-3Flag (OriGene). The plasmid encoding 3xMyc-tagged human MKRN4 was generated by synthesizing DNA and subcloning into the vector pCMV-3Tag-9 (Agilent Technologies). GLK 3'-UTR reporter plasmid (#HmiT055189; GLK 3'-UTR-Luc) and Secrete-Pair™ Dual Luminescence Assay kit were purchased from GeneCopoeia. The reporter plasmids of GLK 3'-UTR (T635C) and GLK 3'-UTR (A644C) were generated by mutating the indicated nucleotide thymine-to-cytosine and adenine-to-cytosine, respectively, on the GLK 3'-UTR-Luc plasmid. The plasmids encoding GLK (A410T), GLK (K650R), GLK (K526R), GLK (K550R), GLK (K620R), GLK (G78V), GLK (M90I), GLK (A199T), GLK (F215S), GLK (M220V), GLK (P436L), GLK (A579T), GLK (D634Y), GLK (A648fs), GLK (C675W), GLK (P704Q), and GLK (T875S) variants were generated by mutating the indicated amino acid residues in the 3xFlag-GLK plasmid. The GLK ΔN (A410T) and GLK ΔN (K650R) plasmids were generated by mutating the indicated amino acid residues in the 3xFlag-GLK ΔN plasmid.

Luciferase reporter assays

Luciferase reporter assays for GLK 3'-UTR activity were performed using the Secrete-Pair™ Dual Luminescence Assay kit (GeneCopoeia) according to the manufacturer's instructions.[5] The 1.352-kb GLK 3'-UTR-driven gaussia-luciferase reporter plasmid containing the secreted alkaline phosphatase (SEAP) internal control was transfected into 5×10^6 Jurkat T cells. After 24 h, 10 µl T-cell supernatants were collected and then incubated with the gaussia luciferase substrate (100 µl GL-H buffer, GeneCopoeia) for 30 s. For internal control, 10 µl T-cell supernatants were incubated at 65 ˚C for 5 min, then incubated with the alkaline phosphatase (AP) substrate (100 µl AP buffer, GeneCopoeia) for 5 min. The levels of gaussia luciferase (Luc) were normalized to that of AP.

***In situ* proximity ligation assay (PLA) technology**

In situ PLA assays were performed using the Duolink In Situ-Red kit (Sigma-Aldrich) according to the manufacturer's instructions as described previously.[4,6] Briefly, Flag-GLK plus Myc-MKRN4-co-transfected HEK293T cells were fixed and permeabilized. Samples were incubated with anti-Flag (#ARG66332, Arigo biolaboratories) and anti-Myc (clone 9E10) monoclonal antibodies, followed by species-specific secondary antibodies conjugated with oligonucleotides (PLA probes, Sigma-Aldrich). After ligation and amplification reactions, PLA signals from each pair of PLA probes in close proximity[7] (< 40 nm; MKRN4-GLK interaction) were visualized as individual red dots and analyzed by Leica DM2500 upright fluorescence microscope. Red dots represent direct interaction signals.

Immunoprecipitation and immunoblotting analysis

Immunoprecipitation was performed by incubation of 1.0 mg protein lysates with 20 µl anti-Flag or anti-Myc Sepharose beads (Merck) at 4 ˚C for 3 h. The immunocomplexes were washed with lysis buffer (50 mM Tris-HCl, 0.2% NP40, 125 mM NaCl, 1.5 mM $MgCl_2$, 5% glycerol, 25 mM NaF, and 1 mM Na_3VO_4) three times at 4 ˚C, followed by boiling in 5x loading buffer at 95 ˚C for 3 min. The immunoblotting analyses were performed as described previously.[4,6]

Liquid chromatography-mass spectrometry and data analysis

For identification of GLK-interacting proteins, immunocomplexes of Flag-tagged GLK were immunoprecipitated by anti-Flag antibody (clone M2; Merck) from lysates of Jurkat T cells transfected with vector or Flag-GLK plasmid. For identification of MKRN4-induced GLK ubiquitination residues, immunocomplexes of Myc-tagged MKRN4 were immunoprecipitated by anti-Myc antibody (9E10; Sigma) from lysates of Jurkat T cells transfected with Myc-MKRN4 plasmid. Protein bands were excised from Instant blue (GeneMark)-stained SDS–PAGE gels. Proteins were digested with trypsin and subjected to LC-MS/MS analyses by LTQ-Orbitrap Elite hybrid mass spectrometer using approaches described previously.[4,6] The peptide data were analyzed by MASCOT MS/MS Ions Search (Matrix Science) under the following condition: peptide mass tolerance, 20 ppm; fragment MS/MS tolerance, 2 Da; allow up to 1 missed cleavage; peptide charge, 2^+, 3^+, and 4^+.

Chuang H-C, *et al. Ann Rheum Dis* 2022; 781:243–254. doi: 10.1136/annrheumdis-2021-221010

Statistical analyses
The normality of each column data was determined by Kolmogorov-Smirnov and Shapiro-Wilk tests using SPSS 25 software. The association of somatic variants with SLE or individual symptoms/treatments was determined by Chi-square test and Fisher's exact test. The statistical significances between two unpaired groups were analyzed using Student's t-test for normally distributed data. All statistical analyses of clinical data were further independently verified by 2 biostatisticians.

Patient and public involvement
Patients or the public were not involved in the design, or conduct, or reporting, or dissemination plans of our research.

References:

1. Hochberg MC. Updating the American College of Rheumatology revised criteria for the classification of systemic lupus erythematosus. *Arthritis and Rheumatism* 1997;40(9):1725.

2. Tan EM, Cohen AS, Fries JF, et al. The 1982 revised criteria for the classification of systemic lupus erythematosus. *Arthritis and Rheumatism* 1982;25(11):1271-7.

3. Lahens NF, Ricciotti E, Smirnova O, et al. A comparison of Illumina and Ion Torrent sequencing platforms in the context of differential gene expression. BMC Genomics 2017;18(1):602.

4. Chuang HC, Tsai CY, Hsueh CH, et al. GLK-IKKβ signaling induces dimerization and translocation of AhR-RORγt complex in IL-17A induction and autoimmune disease. *Science Advances* 2018;4:eaat5401.

5. Tannous BA. Gaussia luciferase reporter assay for monitoring biological processes in culture and in vivo. Nature Protocol 2009;4(4):582-91.

6. Chuang HC, Chang CC, Teng CF, et al. MAP4K3/GLK promotes lung cancer metastasis by phosphorylating and activating IQGAP1. *Cancer Research* 2019;79(19):4978-93.

7. Soderberg O, Gullberg M, Jarvius M, et al. Direct observation of individual endogenous protein complexes *in situ* by proximity ligation. *Nature Methods* 2006;3(12):995-1000.

8. Chuang HC, Lan JL, Chen DY, et al. The kinase GLK controls autoimmunity and NF-κB signaling by activating the kinase PKC-θ in T cells. *Nature Immunology* 2011;12(11):1113-8.

Chuang H-C, *et al. Ann Rheum Dis* 2022; 781:243–254. doi: 10.1136/annrheumdis-2021-221010

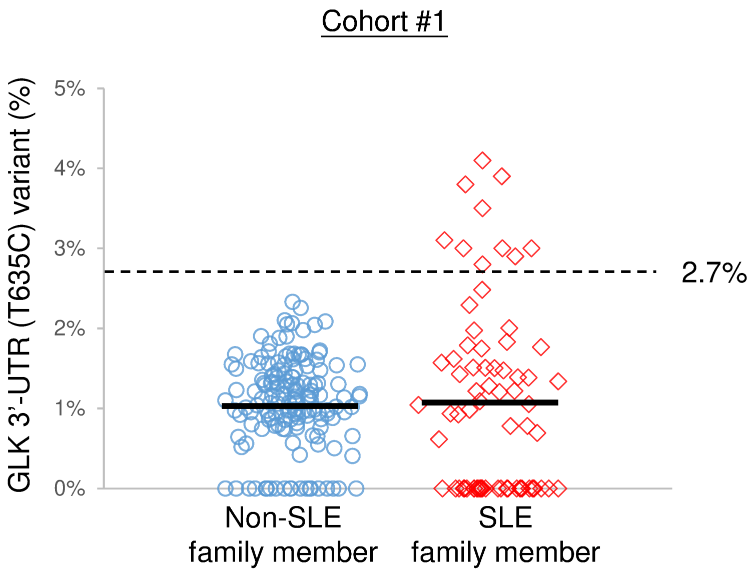

Supplemental Figure S1. The frequencies of GLK 3'-UTR (T635C) variant in SLE family members are increased compared to those of non-SLE family members in Cohort #1. The variant frequency of GLK 3'-UTR (T635C) variant in 157 non-SLE family members and 77 SLE family members of 62 families from Cohort #1. The cut-off value: mean + 3 x standard deviation of 3'-UTR (T635C) variant frequencies in the non-SLE family members was 2.7% (1.06% + 3 x 0.56% = 2.74%).

Chuang H-C, *et al. Ann Rheum Dis* 2022; 781:243–254. doi: 10.1136/annrheumdis-2021-221010

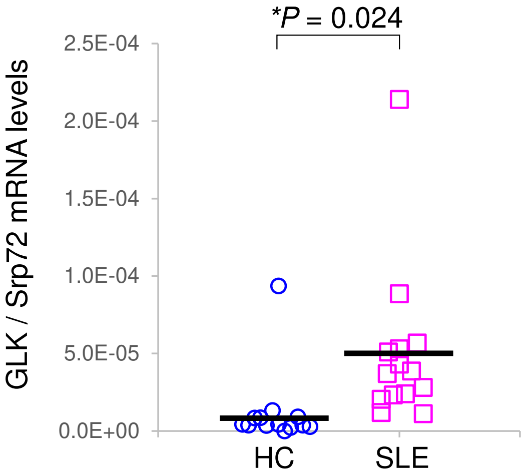

Supplemental Figure S2. MAP4K3/GLK mRNA levels are increased in T cells of SLE patients. Human MAP4K3/GLK mRNA levels in peripheral blood T cells of 14 SLE patients and 13 healthy controls (HC) were analyzed by real-time PCR. These SLE patients and healthy controls were reported previously.[8] The mRNA expression levels of GLK were normalized to signal recognition particle 72 (Srp72) levels. Bars denote means of levels. *, *P* value < 0.05 (two-tailed Student's t-test).

Chuang H-C, *et al. Ann Rheum Dis* 2022; 781:243–254. doi: 10.1136/annrheumdis-2021-221010

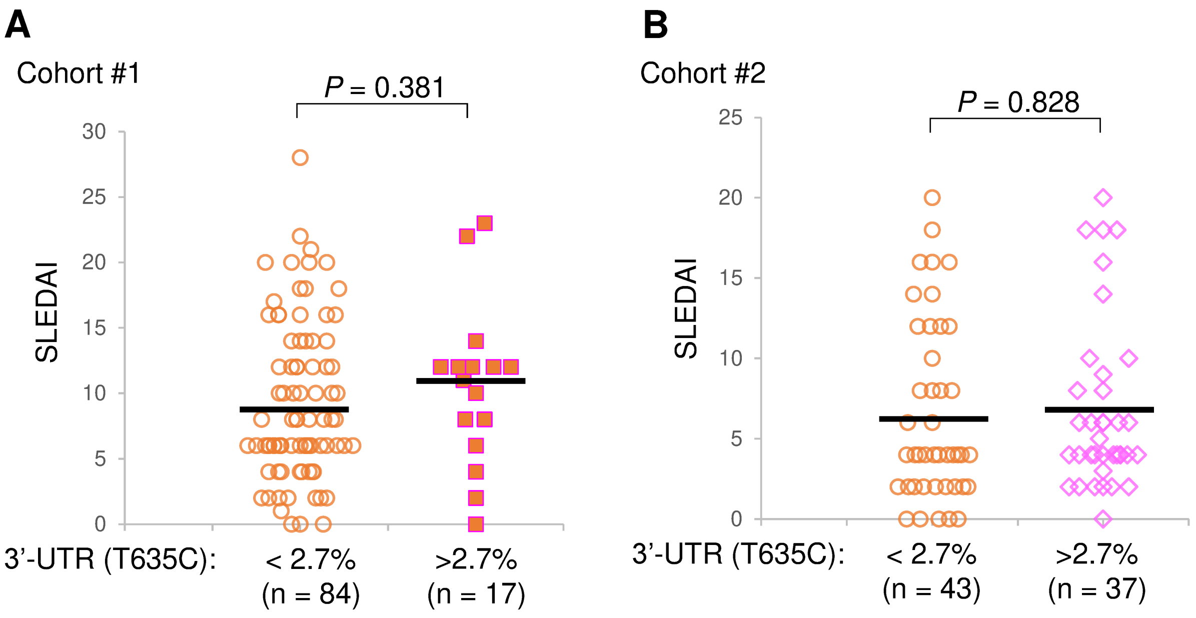

Supplemental Figure S3. GLK 3'-UTR (T635C) variant is not associated with SLEDAI of SLE patients. (**A** and **B**) SLEDAI of Cohort #1 (A) and Cohort #2 (B) SLE patients with a lower (< 2.7%) or higher (> 2.7%) variant frequency. Bars denote means of levels.

Chuang H-C, *et al. Ann Rheum Dis* 2022; 781:243–254. doi: 10.1136/annrheumdis-2021-221010

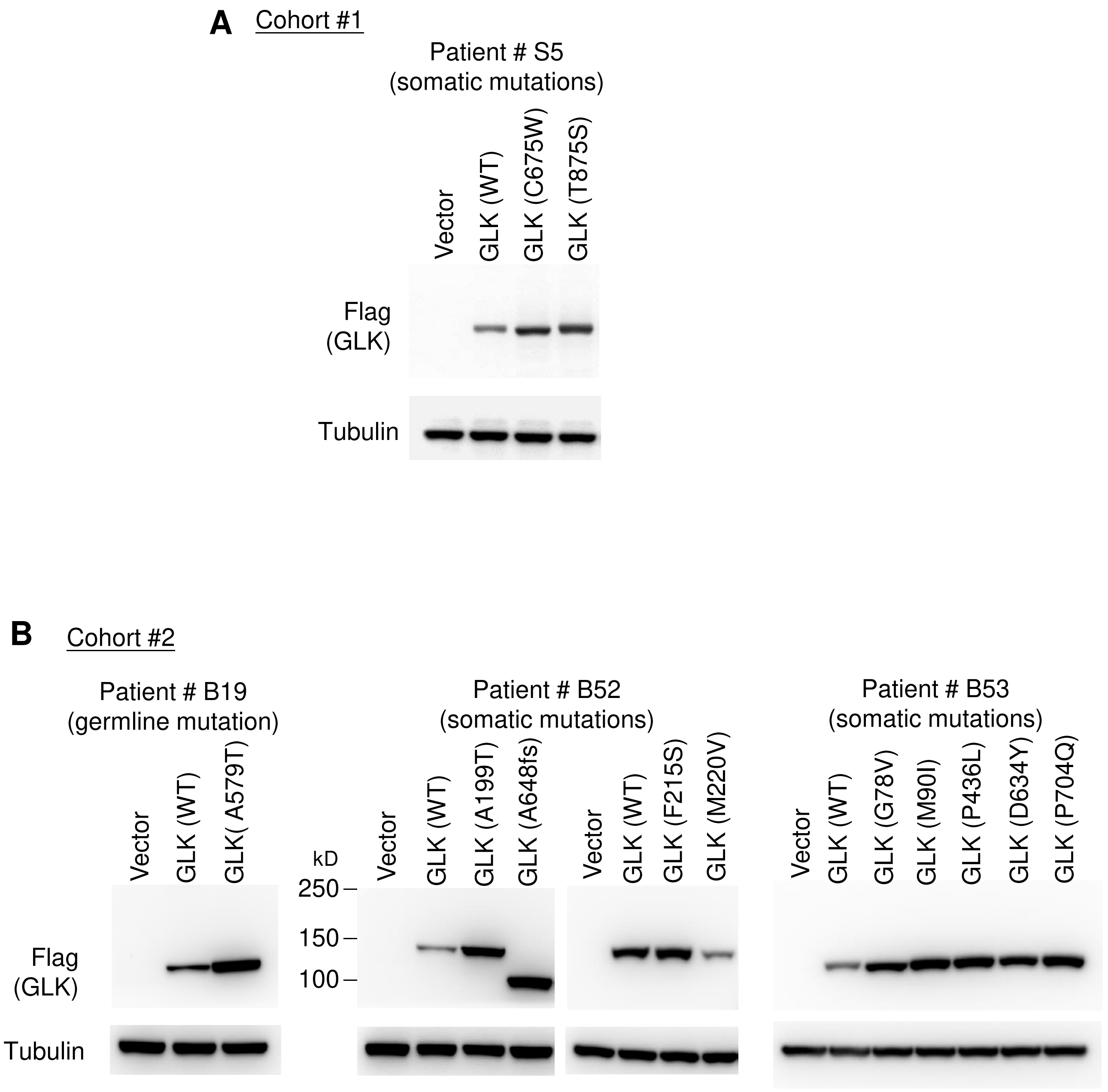

Supplemental Figure S4. Multiple GLK variants enhance GLK protein levels. Panels A shows the immunoblotting of Flag-tagged GLK and tubulin proteins from HEK293T cells transfected with Flag-GLK wild-type (WT), C675W mutant, or T875S mutant plasmid. These GLK variants were identified from Cohort #1. Panels B shows the immunoblotting of Flag-tagged GLK and tubulin proteins from HEK293T cells transfected with Flag-GLK wild-type (WT), A579T mutant, A199T mutant, K648fs mutant, F215S mutant, M220V mutant, G78V mutant, M90I mutant, P436L mutant, D634T mutant, or P704Q mutant plasmid. These GLK variants were identified from Cohort #2. fs, frameshift.

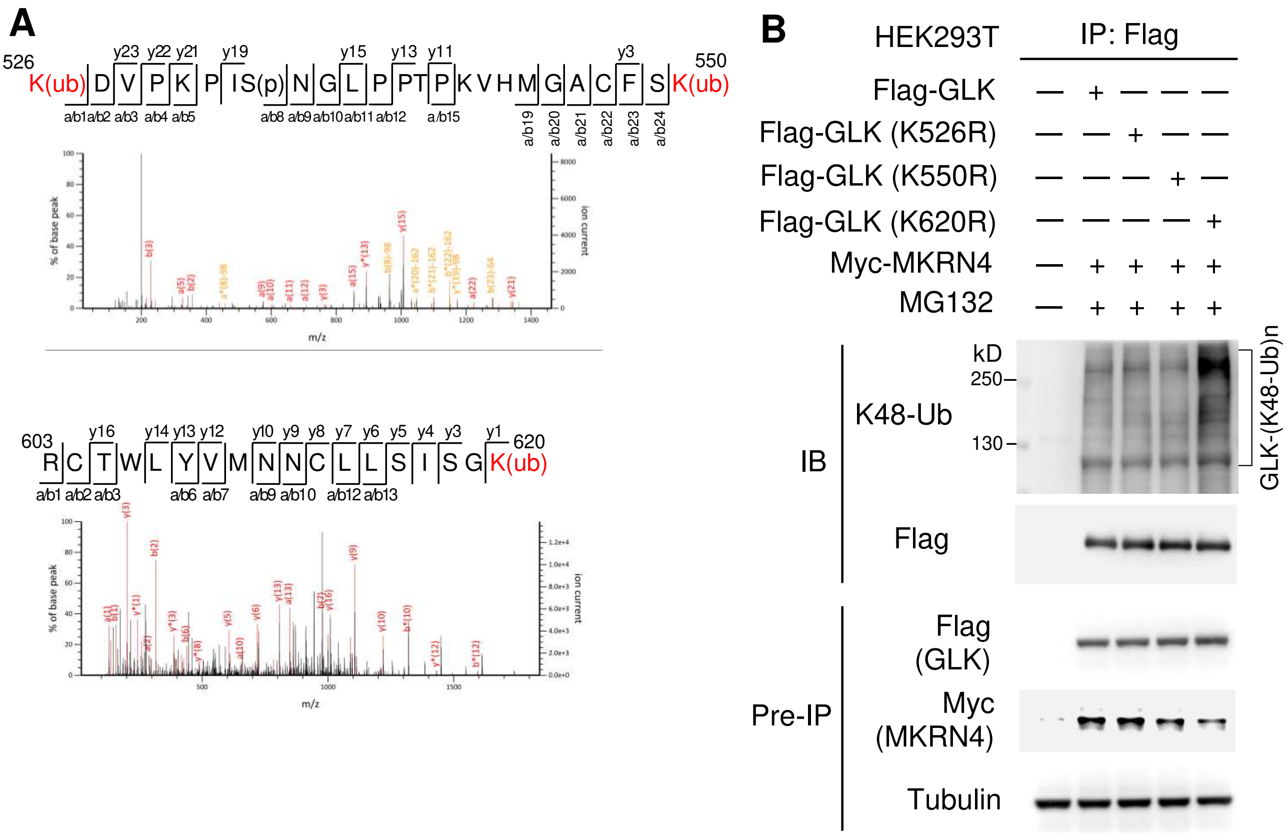

Supplemental Figure S5. Lys526, Lys550, and Lys620 residues are not MKRN4-induced ubiquitination sites on GLK. Panel A shows mass spectrometry analysis of the GLK peptides containing the ubiquitination residue Lys526, Lys550, or Lys620 from the Flag-tagged MKRN4 immunocomplex isolated from the Flag-MKRN4-transfected Jurkat T cells. Panel B shows MKRN4-induced Lys48-linked ubiquitination of GLK. Flag-tagged GLK proteins were immunoprecipitated from the lysates of HEK293T cells co-transfected with Myc-MKRN4 plus Flag-GLK wild-type, K526R mutant, K550R mutant, or K620R mutant plasmid, followed by immunoblotting with either anti-Lys48-linked ubiquitination or anti-Flag antibody. Cells were treated with 25 μM MG132 for 2 h before being harvested.

Chuang H-C, *et al. Ann Rheum Dis* 2022; 781:243–254. doi: 10.1136/annrheumdis-2021-221010

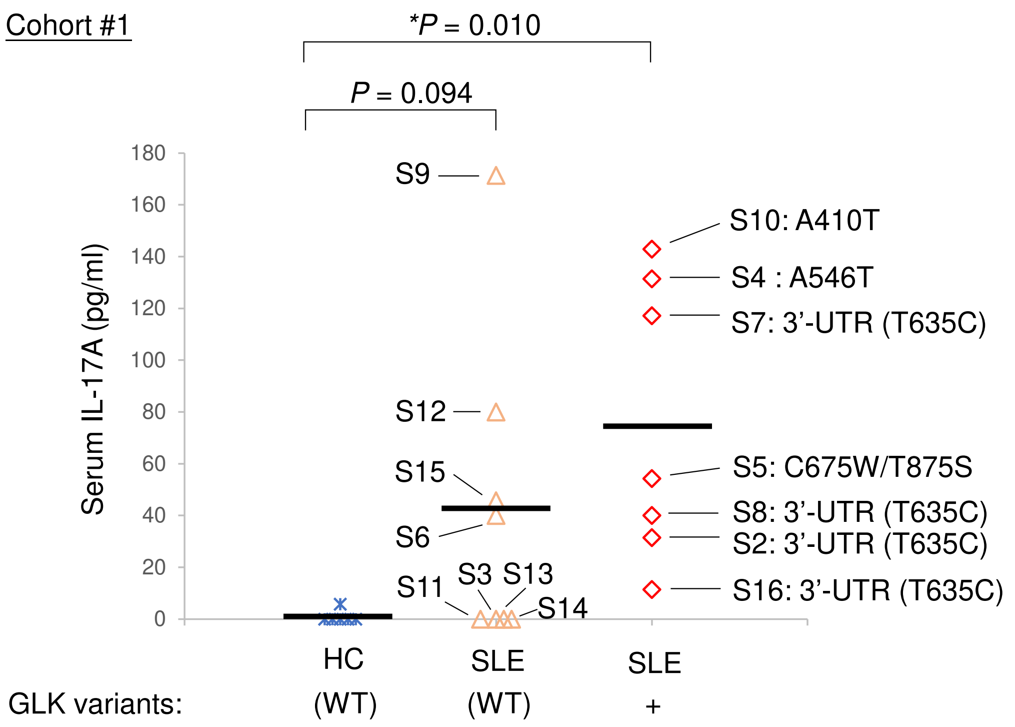

Supplemental Figure S6. SLE patients with GLK variants show high levels of serum IL-17A. ELISA of human IL-17A in the sera of 6 healthy controls, 8 SLE patients without GLK variants, and 7 SLE patients with GLK variants from Cohort #1. Individual SLE (S) ID# and/or GLK variants are indicated. Bars denote means of serum IL-17A levels. *, P value < 0.05 (two-tailed Student's t-test).

Supplemental Table S1. Profile of SLE patients and non-SLE controls in Cohort #1

Cohort #1	SLE (total n = 101)		Non-SLE (total n = 163)	
**Clinical data**	**n or Median**	**% or (Q1-Q3)**	**n or Median**	**% or (Q1-Q3)**
Female, n	n = 86	85.1%	n = 82	50.3%
Age (year-old)	31.0	(26.0-46.0)	40.0	(26.8-55.3)
SLEDAI (score)	8.0	(6.0-14.0)	N/A	
Rashes	n = 68	67.3%	N/A	
Oral ulcer	n = 27	26.7%	N/A	
Arthritis	n = 47	46.5%	N/A	
Serositis	n = 19	18.8%	N/A	
Neuropsychiatic	n = 15	14.85%	N/A	
Nephritis	n = 23	22.77%	N/A	
WBC (count/cumm)	4,795	(3,775-5,632)	N/A	
HgB (g/ml)	12.10	(10.50-13.15)	N/A	
PLT x 1000 (count/cumm)	215.0	(174.0-256.3)	N/A	
Creatinine (mg/dl)	0.90	(0.800-1.020)	N/A	
Serum anti-dsDNA (IU/ml)	231.0	(72.3-455.7)	N/A	
Serum C3 (mg/dl)	79.70	(59.70-93.20)	N/A	
Serum C4 (mg/dl)	13.10	(8.78-17.40)	N/A	

Non-SLE, non-SLE family members of SLE patients and healthy controls; Q1, the first quartile; Q3, the third quartile; N/A, not applicable; WBC, white blood cell; HgB, hemoglobin; PLT, platelet, C3, complement component 3; C4, complement component 4 .

Supplemental Table S2. Comparison of GLK 3'-UTR (T635C) variant between SLE patients (T635C variant frequency > 2.7%) and their non-SLE family members in Cohort #1

Family ID	ID	Diagnosis	GLK 3'-UTR (T635C) frequency	Frequency > 2.7%
F10	1	SLE	0.0%	–
	2	Non-SLE	1.7%	–
	3	Non-SLE	0.0%	–
	4	SLE	3.8%	v
F13	1	SLE	3.5%	v
	2	SLE	0.0%	–
	3	Non-SLE	1.1%	–
	4	Non-SLE	0.0%	–
	5	Non-SLE	1.1%	–
	6	Non-SLE	1.2%	–
	7	Non-SLE	1.2%	–
	8	Non-SLE	1.0%	–
	9	Non-SLE	1.2%	–
F14	1	SLE	2.8%	v
	2	SLE	3.9%	v
	3	Non-SLE	1.8%	–
F15	1	SLE	3.0%	v
	2	SLE	3.0%	v
	3	Non-SLE	1.6%	–
F18	1	SLE	4.1%	v
	2	SLE	2.9%	v
	3	Non-SLE	0.0%	–
	4	Non-SLE	0.9%	–
	5	Non-SLE	0.9%	–
	6	Non-SLE	1.1%	–
	7	Non-SLE	1.2%	–
	8	Non-SLE	2.1%	–
F19	1	SLE	3.1%	v
	2	SLE	0.0%	–
	3	Non-SLE	0.0%	–
F53	1	SLE	3.0%	v
	2	Non-SLE	0.0%	–
	3	Non-SLE	0.0%	–

Supplemental Table S3. Profile of SLE patients and healthy controls in Cohort #2

Cohort #2	SLE (total n = 80)		HC (total n = 87)	
**Clinical data**	**n or Median**	**% or (Q1-Q3)**	**n or Median**	**% or (Q1-Q3)**
Female, n	n = 70	88%	n = 67	77%
Age (year-old)	43.5	(35.8-52.0)	42.0	(35.0-47.0)
SLEDAI (score)	4.0	(2.0-9.3)	N/A	
Rashes	n = 46	58%	N/A	
Oral ulcer	n = 20	25%	N/A	
Arthritis	n = 51	64%	N/A	
Serositis	n = 23	29%	N/A	
Neuropsychiatic	n = 5	6%	N/A	
Nephritis	n = 51	64%	N/A	
WBC (count/cumm)	3,320	(2,537-4,300)	N/A	
HgB (g/ml)	9.50	(8.05-11.05)	N/A	
PLT x 1000 (count/cumm)	144.5	(96.0-191.0)	N/A	
Creatinine (mg/dl)	0.99	(0.885-1.215)	N/A	
Serum anti-dsDNA (IU/ml)	60.00	(9.2-194.5)	N/A	
Serum C3 (mg/dl)	50.55	(39.03-63.225)	N/A	
Serum C4 (mg/dl)	10.00	(5.655-12.95)	N/A	

HC, healthy controls; Q1, the first quartile; Q3, the third quartile; N/A, not applicable; WBC, white blood cell; HgB, hemoglobin; PLT, platelet, C3, complement component 3; C4, complement component 4 .

Supplemental Table S4. Association of GLK 3'-UTR (T635C) variant with organ damage and treatment in Cohort #1

Cohort #1

Symptoms or treatment	GLK 3'-UTR (T635C) variant		
	Low (n = 84)	High (n = 17)	P
Rashes	69.1%	58.8%	0.592
Oral ulcer	33.3%	29.41%	0.975
Arthritis	59.5%	58.8%	1.000
Serositis[f]	26.2%	23.5%	1.000
Neuropsychiatic[f]	21.4%	23.5%	1.000
Nephritis	32.1%	47.1%	0.369
SLEDAI			0.057
no disease activity: 0	0%	5.9%	
mild activity: 1 to 5	15.5%	11.8%	
moderate activity: 6 to 10	34.5%	11.8%	
high activity: 11 to 19	36.9%	58.8%	
very high activity: ≥ 20	13.1%	11.8%	
Cyclophosphamide (Endoxan)	22.0%	33.3%	0.495
MMF[f]	28.0%	26.6%	1.000
Cyclosporine[f]	20.4%	0%	0.100
Azathioprine	46.7%	53.3%	0.838

MMF, mycophenolate mofetil
f, fisher's exact test
Association of GLK 3'-UTR (T635C) with individual symptoms or treatments was determined by two-tailed Chi-Square test (n ≥ 5) or Fisher's Exact test (n < 5).

Chuang H-C, *et al. Ann Rheum Dis* 2022; 781:243–254. doi: 10.1136/annrheumdis-2021-221010

Supplemental Table S5. Association of GLK 3'-UTR (T635C) variant with organ damage and treatment in Cohort #2

Cohort #2

Symptoms or treatment	GLK 3'-UTR (T635C) variant		
	Low (n = 43)	High (n = 37)	P
Rashes	60.5%	54.1%	0.882
Oral ulcer	27.9%	21.6%	0.796
Arthritis	67.4%	59.5%	0.815
Serositis[f]	25.6%	32.4%	0.463
Neuropsychiatic[f]	9.3%	2.7%	0.372
Nephritis	69.8%	56.8%	0.484
SLEDAI			0.306
no disease activity: 0	11.6%	2.7%	
mild activity: 1 to 5	46.5%	51.4%	
moderate activity: 6 to 10	16.3%	29.7%	
high activity: 11 to 19	23.3%	13.5%	
very high activity: ≥ 20	2.3%	2.7%	
Oral cyclophosphamide (Endoxan)	16.3%	18.9%	0.875
Pulse cyclophosphamide (Endoxan)	27.9%	21.6%	0.796
MMF	16.3%	10.8%	0.746
Cyclosporine[f]	6.9%	0%	0.248
Azathioprine	72.1%	64.9%	0.810

MMF, mycophenolate mofetil
f, fisher's exact test
Association of GLK 3'-UTR (T635C) with individual symptoms or treatments was determined by two-tailed Chi-Square test (n ≥ 5) or Fisher's Exact test (n < 5).

Chuang H-C, *et al. Ann Rheum Dis* 2022; 781:243–254. doi: 10.1136/annrheumdis-2021-221010
